# NIR light-assisted phototherapies for bone-related diseases and bone tissue regeneration: A systematic review

**DOI:** 10.7150/thno.49784

**Published:** 2020-09-26

**Authors:** Zhuqing Wan, Ping Zhang, Longwei Lv, Yongsheng Zhou

**Affiliations:** Department of Prosthodontics, Peking University School and Hospital of Stomatology; National Engineering Laboratory for Digital and Material Technology of Stomatology; National clinical Research Center for Oral Diseases; Beijing Key Laboratory of Digital Stomatology, 22 Zhongguancun Avenue South, Haidian District, Beijing 100081, PR China.

**Keywords:** Near infrared light, phototherapy, drug delivery systems, bone-related diseases, bone tissue regeneration

## Abstract

Recently, the rapid development of biomaterials has induced great interest in the precisely targeted treatment of bone-related diseases, including bone cancers, infections, and inflammation. Realizing noninvasive therapeutic effects, as well as improving bone tissue regeneration, is essential for the success of bone‑related disease therapies. In recent years, researchers have focused on the development of stimuli-responsive strategies to treat bone-related diseases and to realize bone regeneration. Among the various external stimuli for targeted therapy, near infrared (NIR) light has attracted considerable interests due to its high tissue penetration capacity, minimal damage toward normal tissues, and easy remote control properties. The main objective of this systematic review was to reveal the current applications of NIR light-assisted phototherapy for bone-related disease treatment and bone tissue regeneration. Database collection was completed by June 1, 2020, and a total of 81 relevant studies were finally included. We outlined the various therapeutic applications of photothermal, photodynamic and photobiomodulation effects under NIR light irradiation for bone‑related disease treatment and bone regeneration, based on the retrieved literatures. In addition, the advantages and promising applications of NIR light-responsive drug delivery systems for spatiotemporal-controlled therapy were summarized. These findings have revealed that NIR light-assisted phototherapy plays an important role in bone-related disease treatment and bone tissue regeneration, with significant promise for further biomedical and clinical applications.

## Introduction

Bone-related diseases such as cancers, infection, and trauma, are common clinical problems that lead to irreparable bone defects, which remains an intractable problem for clinicians, leading to ongoing suffering for patients, and representing a burden to healthcare systems 1. To develop a precise treatment to minimize the adverse influence on the surrounding healthy tissues and to maximize the efficacy of osteogenesis and bone tissue regeneration, selective targeted therapeutic strategies have been investigated 2. Various strategies have been proposed for the targeted therapy of disease regions. A noninvasive technique utilizing external stimulation to control the therapeutic effect on the targeted tissues is an ideal solution 3-5.

Among several external stimuli‑responsive therapy approaches, those using light irradiation have attracted significant attention. The commonly used light for photoresponsive therapies can be generally divided into three subcategories: ultraviolet (UV) (200-400 nm), visible (Vis) (400-700 nm), and near infrared (NIR) (700-1300 nm) lights 3. The choice of different phototherapies in clinical applications is related to the tissue penetration depth of light, which is wavelength-dependent. Compared with UV and Vis lights, NIR light exhibits a higher tissue penetration depth because of its minimum refraction and attenuation by endogenous biomolecules and chromophores (e.g., water, blood, and melanin). For instance, the tissue penetration depth of the rat skin is 7.5 ± 0.5 mm at λ = 705 nm, 6.3 ± 0.5 mm at λ = 633 nm, and only 1.0 ± 0.02 mm at λ = 408 nm, respectively 6. *In vivo* studies in male rats also showed that an 810 nm laser could penetrate 51% through the skull and 40% through the scalp and skull combined in the prefrontal regions. In terms of 660 nm, only 5.8% of light could pass through the scalp plus skull and reach the cortical surface 7. Moreover, the NIR light can be easily controlled in space and time, representing a promising alternative for precisely targeted therapies *in vitro* and *in vivo*. The controlled use of NIR light as an external stimulus is also highly biocompatible, with little toxicity or injurious effects on normal tissues 8. Therefore, NIR light-assisted phototherapy, which realizes effective, noninvasive, precisely controlled therapeutic effects, has attracted increased research attention, leading to multiple solutions in a wide range of fields.

With the rapid development of nanotechnology, a series of NIR light-responsive nanomaterials with specific light absorbance and conversion abilities in NIR light windows have revealed marked potential for phototherapy strategies. Many kinds of nanomaterials, such as carbon-based nanomaterials, metal nanostructures, metal sulfides or oxides, and other organic or inorganic nanostructures, have attracted interest because of their NIR light-responsive properties 9. These NIR light‑responsive nanomaterials have been widely exploited not only for disease diagnosis, but also for photothermal therapy (PTT), photodynamic therapy (PDT), and drug delivery systems because of their good photostability, efficient photothermal conversion ability, and minimal toxicity to normal cells and tissues. One commonly used phototherapy strategy, PTT, directly utilizes photoabsorbing agents to convert the photo energy of the penetrating NIR light into thermal effects, which can be used to ablate nearby abnormal tissues, with minimal damage to normal cells. Contrastingly, during PDT, photosensitizer molecules can generate reactive oxygen species (ROS) under NIR light irradiation, which have a targeted phototherapeutic effect on pathological tissues 2, 8. The local hyperthermia and high ROS concentration have shown stable anticancer performance *in vitro* and *in vivo* 10, 11. Furthermore, NIR light-responsive drug delivery systems have been proposed based on the photothermal and photodynamic effects of nanomaterials. These NIR light-responsive drug delivery systems are able to trigger the controlled release of the encapsulated therapeutics to enhance their therapeutic efficacy 12.

As a noninvasive spatiotemporal controlled treatment technology, NIR light‑assisted phototherapy has played an important role in clinical therapies of many diseases. To date, existing published reviews have mainly summarized the development of NIR light-responsive nanomaterials and their clinical applications for bioimaging and phototherapeutic effect in cancer therapies 11, 13, 14. However, few reviews have discussed NIR light-assisted phototherapies for bone-related diseases and bone tissue regeneration. Recent studies have reported NIR light-induced antibacterial and anti‑inflammatory therapeutic effects for the treatment of bone‑related diseases 15-18. At the same time, several studies have demonstrated that NIR light triggered mild localized heat, and the photoelectronic microenvironment could accelerate bone tissue regeneration 19, 20. Moreover, NIR light-responsive drug delivery systems have not only been used for targeted cancer therapies, but also for the delivery of growth factors for bone tissue regeneration 21, 22. These latest studies have revealed that NIR light‑assisted phototherapy is likely to realize more precise and less invasive therapeutic solutions not only for bone cancers, but also for implant infections, osteochondral inflammation, and bone tissue regeneration. In addition, another photoinduced strategy, photobiomodulation therapy, has been proposed for bone disease treatment and tissue regeneration. However, the above applications of NIR light‑assisted treatment have not yet been reviewed.

Therefore, this article systematically reviews the recent progress of NIR light‑associated phototherapies for bone-related diseases and bone tissue regeneration, intending to reach a general audience comprising not only medical scientists and clinicians, but also material scientists and engineers. Moreover, the current challenges and perspectives within the development of NIR light-assisted phototherapies for further clinical applications are proposed and discussed. A systematic understanding of NIR light-assisted phototherapies would help to achieve the desired therapeutic effects in the treatment of bone-related diseases, providing penetrating insights into precision medicine and regenerative medicine.

## Methods

A primary database collection was constructed for all relevant publications in English (January 1, 1950 - June 1, 2020) based on three databases (PubMed, MEDLINE, and the Web of Science). The following keywords and their combination were used: (NIR OR (near infrared) OR (near infrared light) OR (near infrared laser)) AND bone AND (therapy OR treatment). The keyword search resulted in 1333 initial articles (June 1, 2020). The initial articles were screened based on the following inclusion and exclusion criteria:

Inclusion criteria: 1) Articles focused on bone disease treatment, such as bone cancers, bone infections, osteoarthritis, bone metabolic diseases, bone defect repair, and bone tissue regeneration; 2) only studies using NIR light as the treatment method; and 3) only phototherapies under the NIR light irradiation within the biological window of NIR light (700-1300 nm).

Exclusion criteria included: 1) Duplicate studies; 2) books, reviews, meetings, letters, literature updates, and laboratory protocols; 3) studies using NIR light in the detection and imaging of bone-related diseases (such as NIR spectroscopy for skeletal muscle oxygenation and hemodynamics monitoring, NIR spectroscopy for progressive degeneration of cartilage and bone monitoring, NIR spectrometry for material characterization, and NIR fluorescence for *in vivo* molecule, cell or tissue imaging); 4) articles in languages other than English; and 5) articles without full text (abstract only).

**Figure 1:**
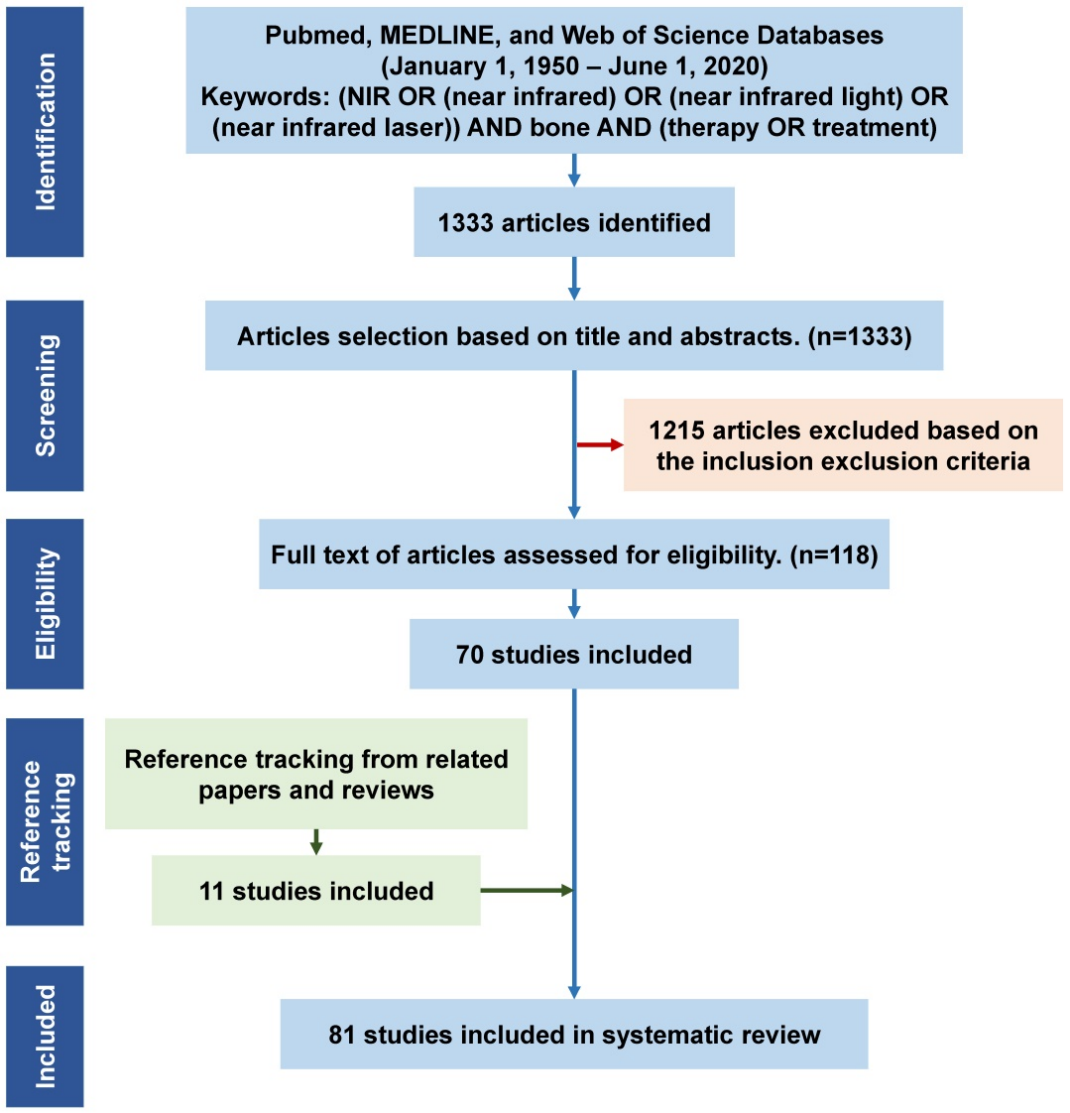
Flowchart for the study screening and selection process, and reasons for inclusion/exclusion. N = number of publications. NIR, near infrared.

Titles or abstracts of the initially retrieved articles were evaluated for inclusion. Each article was reviewed by two researchers independently. When the title and abstract could not be excluded, the full text of the article was evaluated. This resulted in 71 articles fulfilling the eligibility criteria. In addition, reference tracking of the included publications was completed and all the relevant articles were checked and added. Finally, a total of 81 articles were fully examined for discussion in this systematic review (**Figure 1**).

## Results

### Literature search output

Among the 81 included studies, PTT and PDT were used in 39 and 3 studies, respectively, while 11 studies introduced a combined use of PTT and PDT for the treatment of bone-related diseases. NIR light-assisted phototherapies have been used to treat different bone-related diseases (44 studies). The most common types of diseases were osteosarcoma (19 studies), bone metastasis of breast cancer (6 studies), implant‑related infections (8 studies), rheumatoid arthritis (3 studies), and osteoarthritis (1 study). Meanwhile, NIR light-assisted phototherapies have been used to accelerate bone tissue regeneration (13 studies). Twelve of these studies focused on NIR light‑responsive delivery systems to trigger the spatiotemporal controlled release of drugs or molecules to enhance the therapeutic effects to treat bone-related diseases.

**Figure 2:**
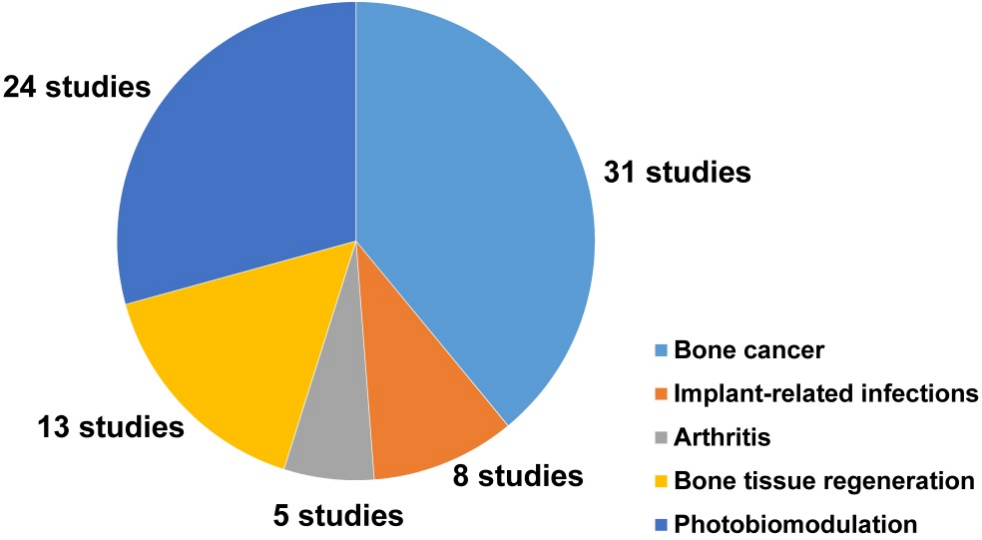
A pie chart representing the number and distribution of different applications of NIR light-assisted phototherapies in the included articles. NIR, near infrared.

Moreover, 24 studies reported the photobiomodulation effects of NIR light irradiation upon cells and tissues, including 8 studies that discussed the photobiomodulation effects on stem cells, and 16 studies that focused on the effect of photobiomodulation therapy on bone healing and dental treatment (**Figure 2**).

### Summary of NIR light-responsive nanomaterials and NIR light radiation conditions

**Figure 3:**
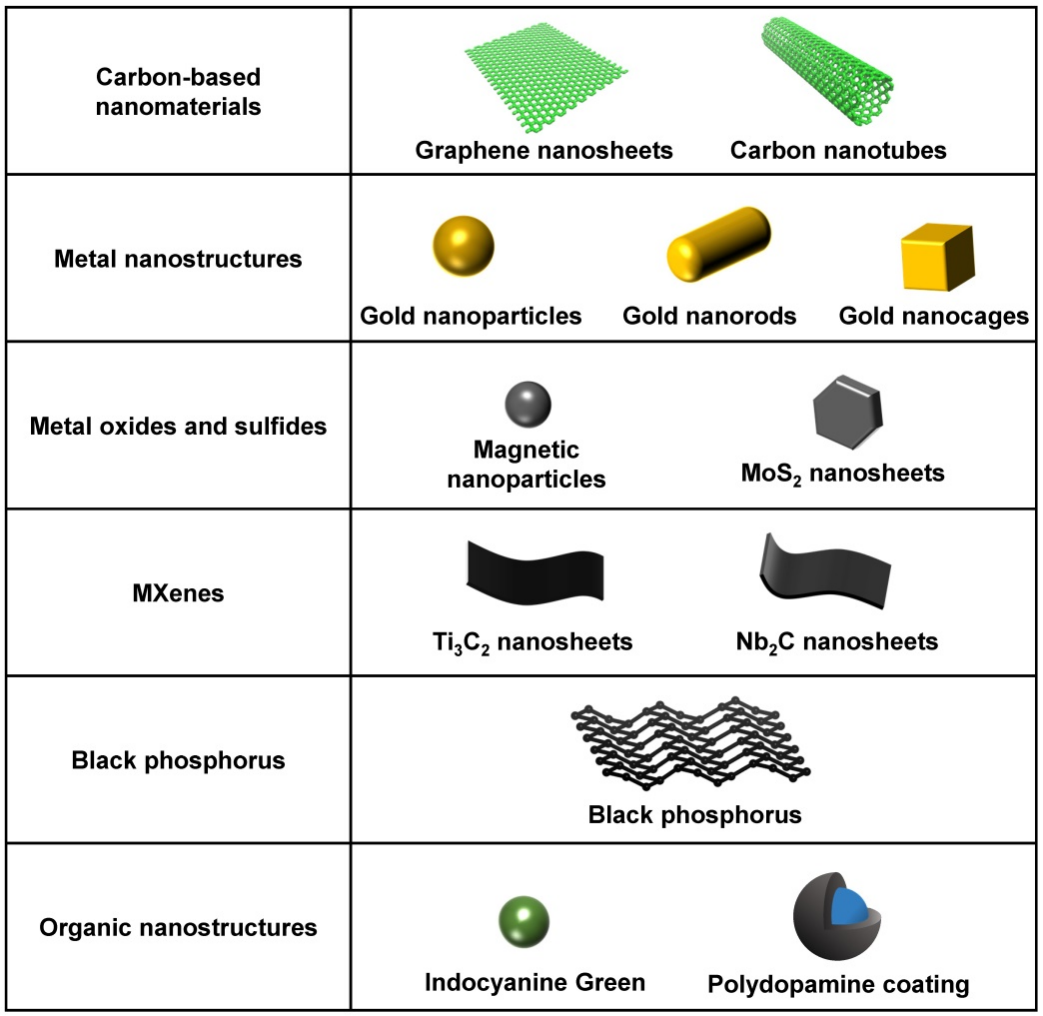
A summary of the current most commonly used NIR light-responsive nanomaterials. NIR, near infrared.

In the 81 included studies, 28 types of photosensitive agents were reported for their superior photothermal and photodynamic performances. These agents comprised a series of biocompatible photothermal transduction agents or photosensitizers based on metal, carbon, transition metal dichalcogenides, polymers, and semiconductors, which have significant NIR light-absorption ability and superior light-responsive capabilities 15, 23-26. They have high photothermal conversion efficiency to generate heat and good photocatalytic characteristics to produce ROS in the NIR region 20, 27, 28. They can also convert NIR light into the secondary local energy, which induces structural changes in drug carriers to trigger drug release 29. The most commonly used NIR light-responsive biomaterials in the included studies were carbon-based nanomaterials (13 studies), metal nanostructures (11 studies), metal oxide nanoparticles (8 studies), metal sulfide nanostructures (9 studies), and black or red phosphorus (7 studies). The current most commonly used NIR light-responsive nanomaterials are summarized in **Figure 3.**

Moreover, to investigate the most suitable radiation conditions, we obtained an overview of the wavelength of the NIR light, the power density and the radiation time used in the different studies and their photothermal or photodynamic effects. The most commonly used window of NIR light for biological application was the NIR-I window (700-1000 nm). NIR light can be generated via many types of available lasers, such as Nd:YAG (neodymium-doped yttrium aluminum garnet) or diode lasers. Most studies (45/81) delivered the NIR light irradiation via an 808 nm diode laser module with fiber coupling. By contrast, phototherapies using NIR coherent (lasers) or noncoherent infrared light (light-emitting diodes, LED) have been investigated for their photobiomodulation effects on stem cells and bone tissue regeneration. Eighteen studies reported the photobiomodulation effect of low level or high frequency NIR laser therapy, while six studies investigated the effect of NIR LED photobiomodulation on cells and tissues.

## Discussion

The results of the literature search showed NIR light-assisted phototherapy exhibited great potential for the precise and controlled treatment of bone-related diseases and bone tissue regeneration because of its deep tissue penetration ability, high spatiotemporal resolution, and the unique biocompatibility of NIR light irradiation. Compared with other traditional therapy strategies, these phototherapies are considered as more minimally invasive, easily controlled, and precise to achieve the desired therapeutic effects and avoid damage to normal tissues. In this systematic review, these NIR light-assisted treatment strategies for bone-related disease and bone tissue regeneration are summarized, based on their mechanisms and applications.

### NIR light-assisted phototherapy for bone cancers

Bone cancers generally refer to malignant bone tumors, including autologous skeletal system cancers (such as osteosarcoma and chondrosarcoma) and bone metastasis of cancers from other tissues (primary cancers in the lung, breast, and prostate) 30, 31. Compared with traditional cancer radiotherapy strategies, NIR light-assisted targeted phototherapies, inducing PTT and PDT, mainly occur in cancer tissues and cause no obvious damage to normal tissues, thus minimizing adverse side effects 32-34. PTT can exploit a local heating effect to realize the ablation of cancer tissues by combining light-absorbing materials with NIR light irradiation. The use of NIR light as an external stimulus allows for remote and high spatiotemporal control of local heating, leading to irreversible destruction of cancer tissue 35. Hyperthermia over 50 °C, followed by NIR light irradiation, can cause irreversible DNA and protein denaturation in cancer regions 36. Contrastingly, in PTT, the photosensitizer enters an excited state after NIR light radiation and generates various ROS from molecular oxygen. This NIR light‑induced release of ROS in PDT can kill cancer cells and damage the cancer vasculature, depriving the cancer of oxygen and nutrients 12, 37.

**Figure 4:**
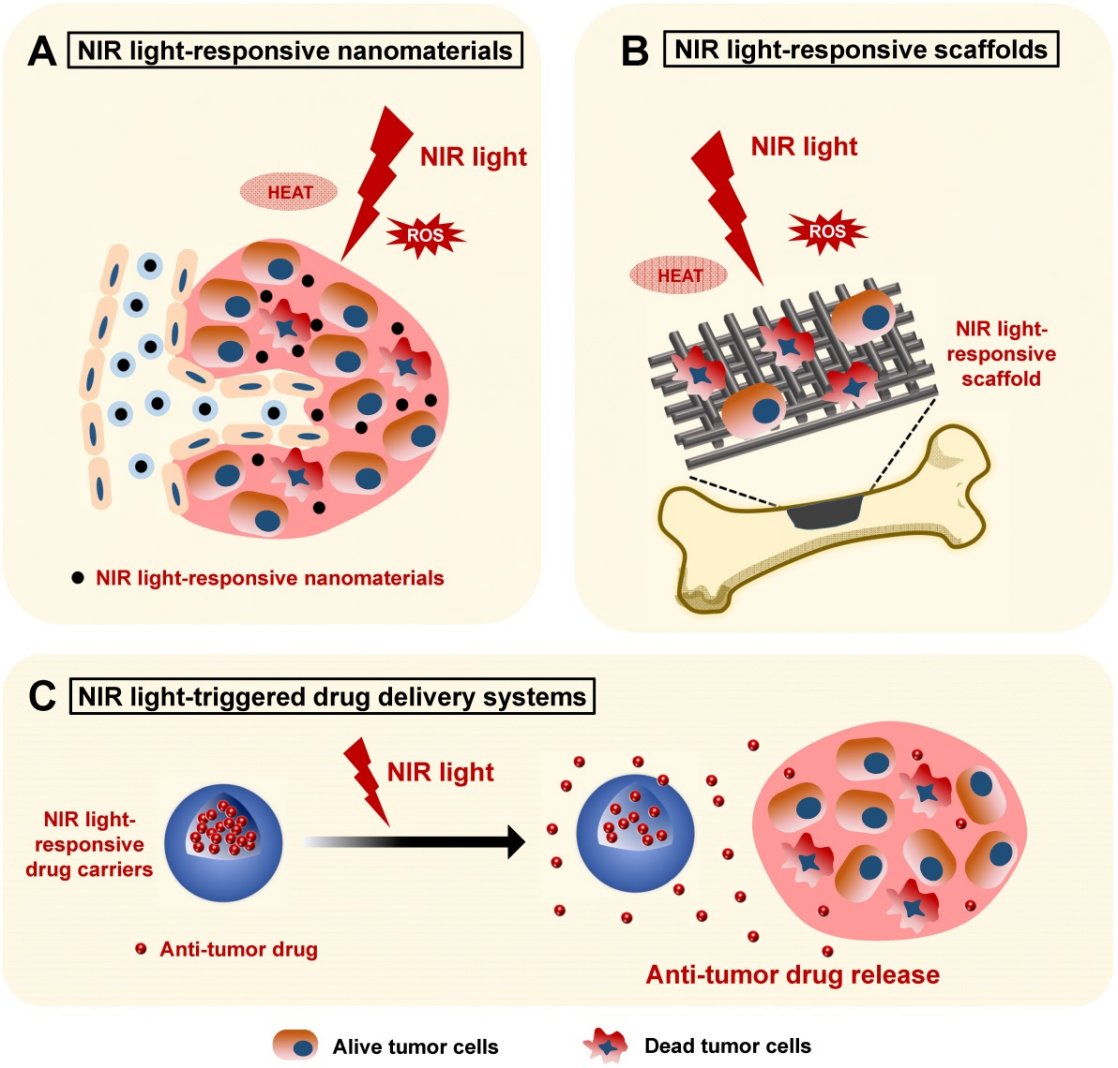
Schematic illustration showing the applications of NIR light-responsive nanomaterials, NIR light-responsive bone scaffolds, and NIR light-triggered drug delivery systems for bone cancer therapy. The NIR light-responsive nanomaterials (**A**) and scaffolds (**B**) with the specific features of highly efficient photothermal conversion and controllable ROS release provide an effective biomaterial platform for the phototherapy of bone cancers. **C.** The NIR light-triggered drug delivery systems can realize controlled release of the encapsulated anti-tumor drugs for bone cancer chemotherapy. NIR, near infrared; ROS, reactive oxygen species.

With the rapid development of nanotechnology, a series of NIR light-responsive nanomaterials have emerged and shown great advantages for efficient bone cancer phototherapy by serving as photothermal agents for PTT or as photosensitizers for PDT. 38-40. According to the different administration methods, there are three main applications of these NIR light-responsive nanomaterials for bone cancer therapy. Firstly, these nanomaterials could be injected intravenously. The prolonged blood circulation time and the targeted tumor accumulation effects of these nanomaterials make highly efficient* in vivo* PTT and PDT possible, offering promising options for the treatment of the primary bone cancers or problematic cancer bone metastasis from other tissues 41. However, the direct intravenous injection of nanomaterials might cause *in vivo* toxicity. The biosafety of these NIR light-responsive nanomaterials should be confirmed by testing normal hematology parameters to minimize the potential side effects 42. Secondly, these NIR light-responsive nanomaterials can be integrated into scaffolds for combinatory bone cancer therapy and tissue regeneration. Considering that a few bone tumor cells inevitably remain around the bone defect after surgical intervention, the implanted bifunctional scaffolds could exhibit not only prominent photothermal and photodynamic performances, but also outstanding bone regeneration capabilities, which provide optimal alternatives for the treatment of large bone tumors and tumor-initiated bone defects 31, 43-45. Thirdly, these NIR light-responsive nanomaterials could also be integrated into NIR light-triggered drug release systems, exhibiting versatility via their precise and controlled release of anticancer drugs 12. NIR light-absorbing nanomaterial‑doped drug-carriers could exhibit outstanding photothermal and photodynamic therapeutic effects, as well as superb anticancer drug encapsulation efficiency, through intravenous administration, serving as promising candidates for bone cancer therapies 46. In this section, we will introduce these three applications of NIR light-assisted phototherapy for bone cancers based on three different administrations. The mechanisms of these applications are shown in **Figure 4.**

#### NIR light-responsive nanomaterials in the phototherapy of bone cancers

The efficiency of anticancer phototherapy depends on the photothermal and photodynamic performances of the employed NIR light-responsive nanomaterials. Several nanomaterials, such as carbon-based nanomaterials 47, metal nanoparticles 48, and ceramic-based nanomaterials 32 are being developed for bone cancer phototherapies because of their efficient photothermal conversion function under NIR light irradiation after direct intravenous administration. A series of carbon-based nanomaterials have been widely used in NIR light-assisted phototherapies because of their excellent photothermal performances under the NIR light irradiation. For example, nanosized graphene oxide (GO) has been used as a localized, minimally invasive therapeutic approach for osteosarcoma therapy by inducing hyperthermia-medicated membrane damage of cancer cells under 808 nm NIR irradiation 47. Similarly, multi-walled carbon nanotubes (MWNTs) can also result in an efficient hyperthermia under 808 nm NIR irradiation and could remarkably suppress the growth of tumors, reducing the amount of cancer-induced bone destruction 49. At the same time, metal nanoparticles, such as the classical platinum nanoparticles, could display enhanced accumulation at cancer-bearing bone lesions and inhibit the growth of bone cancer via PTT under an 808 nm NIR laser irradiation 48, 50. Jie *et al.* reported a novel kind of oxygen vacancy-rich tungsten bronze nanoparticle (Na_x_WO_3_) against breast cancer osteolytic bone metastasis, which acts via the desirable photothermal effect under NIR laser irradiation 51. This treatment strategy could also inhibit the osteoclastic RANKL and Sclerostin expression from tumor cells, thus further attenuating downstream osteoclastogenesis. Notably, the shapes of these nanoparticles can change their photoresponsive properties, and NIR absorbing nanorods and nanocages have been preferred for *in vivo* applications 4. The heat released by these nanoparticles under NIR light irradiation would efficiently kill cancer cells via photothermal ablation. Meanwhile, these nanosized particles can be cleared efficiently via the kidneys, decreasing their long-term toxicity in clinical applications 50.

Alternatively, the tissue penetration efficacy of NIR light would also influence the efficiency of anticancer phototherapies. The biological window of NIR light can be divided into NIR-I (700-1000 nm), and NIR-II (1000-1300 nm) biowindows 32, 52. To date, the NIR-I biowindow has been more commonly used in phototherapies because most kinds of NIR light-responsive nanomaterials have exhibited favorable photothermal and photodynamic properties in the NIR-I biowindow. Considering that the tissue penetration depth of light increases with increasing wavelength, the NIR-II biowindow exhibits deeper tissue penetration compared with the NIR-I light. It would be helpful to develop efficient photoresponsive nanomaterials with high NIR-II absorbance 35, 42. Recently, a new family of two dimensional (2D) nanomaterials, named MXenes, have generated great interest because of their unique physiochemical performance and excellent photothermal conversion efficiency 53. These 2D nanomaterials, which comprise transition metal carbides, nitrides, or carbonitrides, have displayed highly efficient *in vivo* photothermal performance in both the NIR-I and NIR-II windows 54. For example, Lin's group reported a 2D niobium carbide (Nb_2_C), a novel kind of MXene, with an extraordinarily high efficiency of photothermal conversion for bone tumor ablation 32. They revealed its highly efficient *in vitro* and *in vivo* photothermal cancer therapies in both NIR-I (36.4%) and NIR-II (45.65%) biowindows. Similarly, hybrid carbon dots (CDs)/WS_2_ heterojunctions have also been reported for NIR-II enhanced PTT of osteosarcoma. These CDs/WS_2_ heterojunctions exhibited improved cancer targeting ability after intravenous injection and efficient photothermal conversion under the 1064 nm laser irradiation. The deep tissue penetration ability in the NIR-II biowindow means that the satisfying ablation of cancer tissues can be realized at relatively low laser exposure, even when being covered by a 10 mm thick additional tissue. Although the NIR-II biowindow has attracted great attention for tumor xenografts, to date, it has rarely been used for bone cancer therapies, mainly because of a lack of suitable photosensitive agents with desirable strong absorption and high efficiency of photothermal conversion in the NIR-II biowindow 32, 55-57. More effort should be made to develop efficient light-responsive nanomaterials with high NIR-II absorbance to broaden the application prospects of NIR light-assisted phototherapies for bone cancer.

Meanwhile, nanomaterials can serve as an effective photosensitizer to generate ROS after NIR light irradiation for photodynamic therapy in bone cancer therapy. Pd‑bacteriopheophorbide has been reported as a photosensitizer that induces PDT, leading to hypoxia, necrosis, and eradication of prostate cancer bone metastasis 41. This photosensitizer could target the tumor vasculature after direct intravenous administration and is cleared rapidly from the circulation within a few hours. Raucci *et al*. 58 proposed ultrathin 2D black phosphorous (BP) as a promising candidate for osteosarcoma therapy because of its photodynamic effect. 2D BP could inhibit the metabolic activity of osteosarcoma cells and induce the proliferation and osteogenic differentiation of human preosteoblast cells *in vitro*, thus playing important roles in anticancer therapies and regenerative medicine. The nanomaterials and the different radiation conditions used in current bone cancer phototherapies are summarized in **Table 1.** These nanomaterials show great promise for bone cancer therapy and bone tissue engineering.

#### NIR light-responsive scaffolds for the phototherapy of bone cancers

Massive cancer-induced bone defects and residual cancer cells are two critical problems in the prognosis of bone cancers. It is essential to fabricate multifunctional scaffolds endowed with the ability to eradicate cancer cells and remodel bone tissue simultaneously during bone cancer therapy 31. In the present review, a series of strategies have been proposed to combine NIR light-responsive nanomaterials with 3D printed scaffolds for bone defect repair after the resection of cancer tissue. These 3D printed scaffolds, such as bioactive glass (BG) scaffolds and tricalcium phosphate (TCP) scaffolds are commonly used scaffolds for bone tissue engineering. The combination of these bioactive BG scaffolds with NIR light-absorbing agents could endow the scaffolds with excellent photothermal and photodynamic therapeutic effects after implantation into the tumor-induced bone defects. Furthermore, the implanted bioactive scaffolds could also facilitate new bone formation by stimulating the osteogenic differentiation of bone mesenchymal stem cells (BMSCs) 59. The favorable osteogenic ability of these scaffolds is mainly attributed to their interconnected macropore structure for nutrient transportation and the gradual release of elements (such as Ca, P, and Si) during the degradation process 43, 59. Currently, a series of bifunctional scaffolds have been developed based on the constantly emerging NIR light-responsive nanomaterials. The main methods and conclusions of relevant studies are listed in **Table 2.**

The classical metal nanomaterials have generated great interest because of their excellent photothermal conversion performance in anticancer therapies. It has been reported that bismuth-doped BG scaffolds displayed high photothermal conversion efficiency to induce hyperthermia for osteosarcoma treatment 60. Meanwhile, multiple kinds of metal oxide nanoparticles have been used as photoabsorbers to kill cancer cells. Zhang's groups have reported a black TiO_2_ nanoparticles-coated Ti6Al4V implant for the reconstruction of large bone defects followed by surgical resection of bone cancers 61. The black TiO_2_ nanoparticle coating endowed the implant with good biocompatibility, strong NIR light absorbance, and efficient photothermal conversion ability under 808 nm laser irradiation. Similarly, magnetic iron oxide nanoparticles, such as Fe_3_O_4_ nanoparticles and M-type ferrite nanoparticles (SrFe_12_O_19_), could serve as photothermal agents in scaffolds. These scaffolds could exhibit photothermal anticancer therapeutic abilities and enhanced bone regeneration simultaneously 62-64. Meanwhile, metal-organic frameworks (MOFs), such as copper coordinated tetrakis (4‑carboxyphenyl) porphyrin (Cu-TCPP) also have strong NIR light absorption 65. The released bioactive ions of the Cu-TCPP combined with TCP scaffolds can simultaneously stimulate osteogenesis and angiogenesis in the bone defect. Moreover, a copper-based CuFeSe_2_ nanocrystal, a kind of semiconductor, has been reported as a new class of efficient photothermal agent. By functionalization with the CuFeSe_2_ nanocrystals, 3D printed BG scaffolds exhibited excellent photothermal performance under 808 nm laser irradiation 43. Another kind of PTT agent, MoS_2_ nanosheets, has also been introduced into BG scaffolds for anticancer treatment and bone tissue regeneration 66. Besides these inorganic nanomaterials, a kind of NIR light-absorbing organic charge-transfer cocrystal with great photothermal conversion performance has also been integrated into BG scaffolds to fabricate bifunctional therapeutic implants 45. Additionally, organic NIR light-responsive biomaterials modification, such as dopamine modification, would also provide scaffolds with an excellent NIR photothermal effect 67. These modified bifunctional scaffolds could be implanted precisely into the tumor sites and could induce effective local hyperthermia (over 50 °C) under the NIR light irradiation, which resulted in enhanced tumor cell apoptosis and tumor ablation. After irradiation, the mesenchymal stem cells (MSCs) could migrate to the bone defects gradually and attach, proliferate, and differentiate on the bioactive scaffolds, leading to long-term bone regeneration.

In addition, carbon-based nanomaterials, such as GO and single-/multi-walled carbon nanotubes, offer great promise for cancer photothermal therapies because of their strong NIR light absorbance, excellent photothermal performance and satisfactory cytocompatibility 68-71. It also has been reported that carbon-based nanomaterials can effectively improve bone regeneration by inducing the directional migration and osteogenic differentiation of BMSCs 72, 73. For example, GO‑modified biofunctional tricalcium silicate bone cement and TCP bone scaffolds have been fabricated for cancer therapy and bone tissue regeneration 59, 71. The MWNT-modified scaffold also exhibited high photothermal performance in the treatment of cancer related bone defects 70. Zero dimensional carbon dots are another kind of carbon-based nanomaterial. They can bind specifically to calcified bones and have been used to eliminate osteosarcoma and enhance bone tissue regeneration 68. *In vitro* and *in vivo* studies of these carbon-based nanomaterial-coated scaffolds have successfully demonstrated their bifunctional properties of photothermal therapy and bone regeneration, which could pave the way for the design and fabrication of novel implants with simultaneous bone cancer killing and bone tissue-remodeling capacity.

With the rapid advance of nanotechnology, a series of new nanomaterials, including MXenes and Lanthanide (Ln) nanomaterials, have also been incorporated into bone scaffolds for cancer phototherapies recently. MXene nanosheets, such as 2D Ti_3_C_2_ MXene and Nb_2_C MXene, have unique structural characteristics, including a large specific surface area and adjustable physiochemical properties. They display enriched photothermal and photodynamic therapeutic effects in both the NIR-I and NIR-II biowindows, providing intriguing biomaterial platforms for the diversified treatment of bone cancers 31, 44. LaB_6_, a chemical compound of La and B elements, also shows efficient NIR photothermal conversion ability. The LaB_6_-modified TCP scaffold is an ideal option for both the ablation of bone cancers and the repair of defect regions 74. The emergence of these novel photothermal agents has provided the possibility for the development of NIR light-responsive scaffolds, offering new avenues to construct highly efficient therapeutic platforms for bone caners.

#### NIR light-triggered drug delivery systems for bone cancer chemotherapy

Controlled drug delivery systems exhibit an enhanced therapeutic efficiency as well as reduced systemic toxicity compared with conventional bone cancer chemotherapies. Photothermal (generation of heat) and photodynamic (production of ROS) mechanisms could be used to break, isomerize, or rearrange molecules and achieve drug release. After reaching the cancer tissues, the drug delivery systems can be triggered by NIR light to realize controlled release of the encapsulated therapeutics 12. The energy of NIR light radiation could be converted into heat in photothermal systems. This heat conversion can change the physicochemical properties of thermoresponsive agents in drug delivery systems, resulting in drug release 12, 75. In the photodynamic-induced release system, the photosensitizer generates singlet oxygen (^1^O_2_) from molecular oxygen after NIR light radiation and mediates the PDT. The highly active ^1^O_2_ interacts with the specific molecule in the drug delivery system, leading to a structural change that triggers drug release 12, 75.

Considering that the well-distributed porous structure of mesoporous silica nanoparticles (MSNs) is promising for drug loading and encapsulation, a series of NIR light-absorbing agent-doped MSNs have been designed for photochemotherapy of bone cancer. For example, Lu *et al*. 46 designed novel bismuth sulfide (BS)@MSNs to deliver doxorubicin (DOX), a classical anticancer drug, to treat osteosarcoma. The outstanding photothermal effect of the BS nanoparticles not only controlled the release of DOX, but also induced cell death, exerting a combined bone-targeted chemothermal therapeutic effect. Xue's group also engineered a biodegradable polydopamine (PDA) coated bioactive glass nanoparticle platform for on-demand NIR light-triggered anticancer drug (DOX) release and photothermal ablation of bone cancers 76. Similarly, Sun's group have designed a gold nanorod-decorated mesoporous silica nanoparticle to deliver zoledronic acid (ZOL), which exhibits strong bone affinity and efficient anticancer abilities for breast cancer bone metastasis 77.

In addition, mussel-inspired PDA nanoparticles also possess efficient photothermal conversion and intrinsic drug anchoring abilities 78, 79. They have been used widely as versatile carriers for anti-tumor drug delivery in the photochemotherapy of bone cancer. For example, Wang's groups have designed alendronate (ALN)-anchored PDA nanoparticles to release the anticancer drug, 7-ethyl-10-hydroxycamptothecin 80. Similarly, injectable chitosan hydrogels containing PDA-decorated hydroxyapatite (HAp) nanoparticles could realize localized controlled and sustained release of cisplatin when exposed to NIR light 81. Moreover, a kind of dual responsive drug delivery system, which could be triggered by both NIR laser irradiation and acidic stimulus, has been developed for bone cancer therapy. Gu *et al*. 82 reported a bovine serum albumin-bioinspired iridium oxide nanosystem for chemophotothermal therapy of osteosarcoma. This nanosystem displayed both NIR light and pH-responsive DOX release profiles *in vitro*, which enhanced the therapeutic anticancer effect. The above anticancer drug carrier systems could be administrated through intravenous injection and are mainly distributed to, and accumulated in, bone cancer tissues, displaying efficient photochemotherapeutic performance under NIR laser irradiation. The radiation parameters of NIR light-induced phototherapies, as well as the encapsulation and release efficiencies of these anticancer drug delivery systems, are summarized in **Table 3.**

### NIR light-assisted phototherapy for bone infection and inflammation

**Figure 5:**
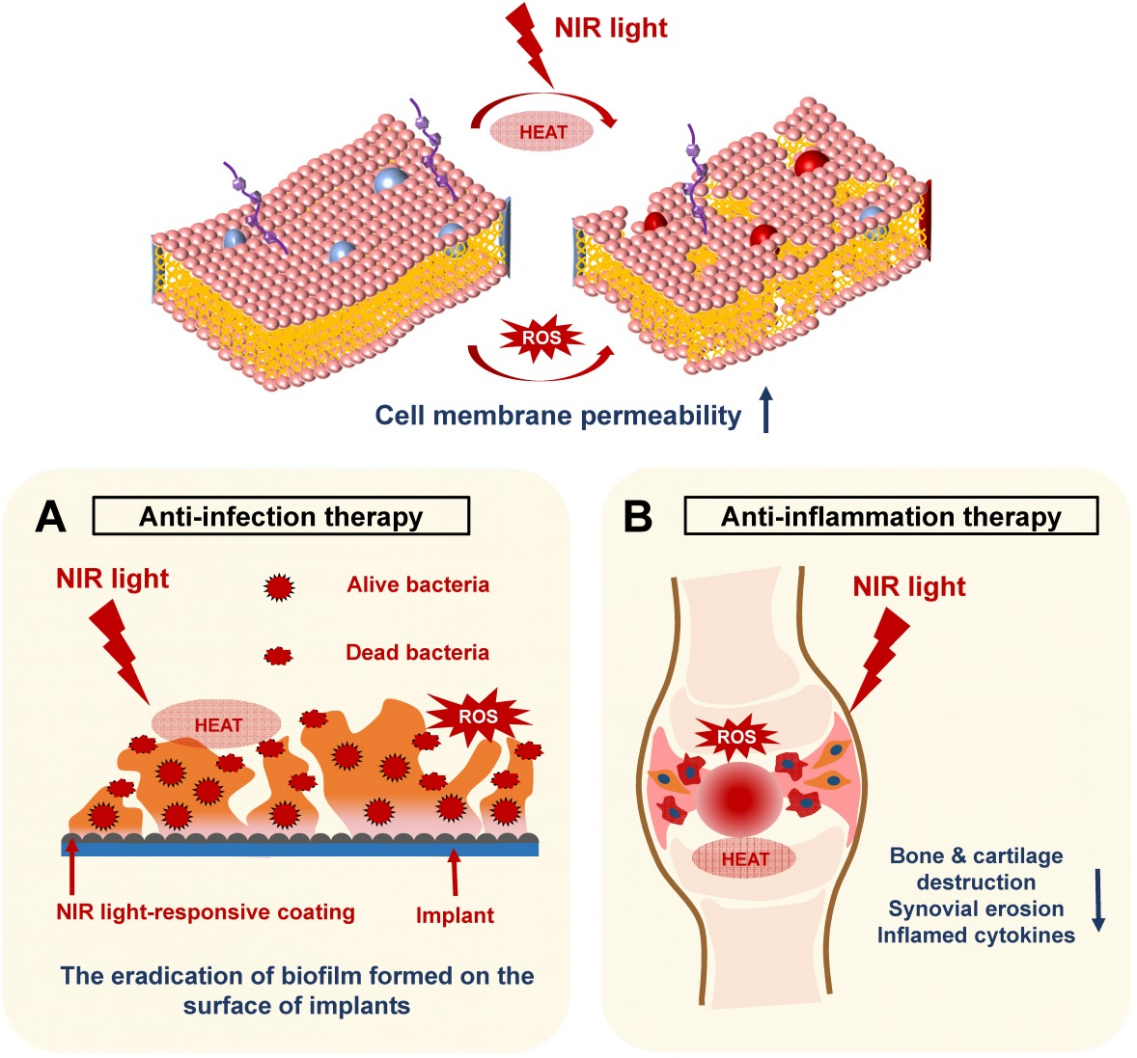
Schematic illustration revealing the mechanisms of NIR light-assisted phototherapy for bone infection and inflammation. **A.** NIR light-induced hyperthermia increases the bacterial membrane permeability under ROS stimulation, accelerating the eradication of the biofilm formed on the surface of the implants. **B.** The NIR light can penetrate the inflamed joints and generate local heat and ROS to eliminate inflamed tissues, decreasing the generation of inflammatory cytokines, synovial erosion, and tissue destruction. NIR, near infrared; ROS, reactive oxygen species.

In recent years, NIR light-assisted phototherapy has been recognized as a promising strategy, not only for anticancer therapy, but also for antibacterial and anti‑inflammation therapies 15, 58, 83. The NIR light-responsive nanomaterials could be coated onto the bone implants, exhibiting outstanding photothermal and photodynamic therapeutic effects to destroy bacterial integrity or biofilm structure via hyperthermia and ROS generation 16. Meanwhile, NIR light-responsive nanomaterials can be directly injected into the inflamed joints, inducing local heat under NIR irradiation and delivering ROS simultaneously to remove inflamed tissues 83. Therefore, the current applications of NIR light-assisted phototherapies for classical implant-related bone infections, as well as common chronic arthritis, will be introduced and summarized in this section (**Figure 5**).

#### NIR light-assisted phototherapy for implant-related infections

Massive bone defects caused by bone cancers, infections, fractures, and the increasing number of orthopedic diseases have led to a high demand for artificial implants 84. Bacteria‑induced infections of artificial implants and insufficient bone tissue integration are still the main problems after implantation, which could lead to implant failure 85, 86. The implant surface could provide appropriate conditions for bacteria to adhere and proliferate. Once a pathogenic biofilm forms on the implant surface, the host defense and conventional antibiotic treatment cannot work, leading to infections of the surrounding tissues 87, 88. The implants coated with the NIR light-responsive nanomaterials have received increased attention because of their outstanding photothermal and photodynamic therapeutic performances. These implants could exhibit controlled, noninvasive, and nonsurgical antibacterial properties by generating heat and ROS. The antibacterial efficiency reached about 97% for *Staphylococcus aureus* at a hyperthermia of 88.8 °C for 15 min when photothermally triggered by NIR light 28. Moreover, releasing ROS, such as ^1^O_2_, can damage cell membranes and cell walls of bacteria and kill the pathogens 89. However, when using photothermal or photodynamic therapy alone, the excessive temperature caused by the photothermal effect or release of ROS during the photodynamic therapy could damage normal tissue and induce serious side effects. Therefore, it is desirable to combine the two therapies to kill bacteria. A high temperature of 50 °C can facilitate permeability of the bacterial membrane, making it easier to kill bacteria via ROS 27, 28. In addition to efficient antibacterial properties, ideal bone implants should also display good biocompatibility and promote osteogenic capabilities. Based on these mechanisms, several studies have reported NIR light-assisted antibacterial coating on the surface of bone implants to treat implant-related infections. These NIR light-assisted antibacterial strategies are discussed in more detail below and are summarized in **Table 4.**

For example, mussel-inspired PDA is emerging as an excellent photothermal agent because of its good biocompatibility and efficient NIR light-responsive photothermal conversion. It has been reported that photosensitizer-loaded mesoporous PDA nanoparticles, as well as MoS_2_/PDA-arginine-glycine-aspartic acid (RGD) decorations of bone implants, displayed synergistic photothermal and photodynamic effects for antibacterial therapy 16, 17. Similarly, a hybrid BS@Ag_3_PO_4_ coating on bone implants also exhibited good photothermal effects using BS as photocatalyst 90. The integration of Ag_3_PO_4_ also endows the implant with bacteriostatic properties. This NIR light-triggered inorganic hybrid semiconductor heterojunction-decoration system is biocompatible and effective to eliminate biofilms. In addition, red phosphorus has been proven to be an efficient photothermal coating on bone implants because of its good biocompatibility and more effective photothermal ability compared with BP. Its degradation products are mainly phosphate, which plays an important role in bone tissue regeneration 91. Tan's group have reported a red phosphorus-IR780-RGDC (arginine-glycine-aspartic-acid-cysteine)-coated antibacterial titanium (Ti) bone implant 28, 92. The outstanding photothermal effects of red phosphorus and the stable photodynamic effects of IR780 meant that the biofilm could be eliminated efficiently under a safe 808 nm NIR light irradiation at 50 °C, *in vitro* and* in vivo*, without damaging the normal tissues. At the same time, the RGDC decoration on the surface of the Ti implant enhanced the adhesion, proliferation, and osteogenic differentiation of MSCs. These coating strategies on the surfaces of bone implants have provided great promise for clinical orthopedic applications.

Hang *et al*. 27 have reported another dual light coirradiation system, including both 660 nm visible light and 808 nm NIR light, for biofilm elimination and osteointegration of bone implants. They designed a biocompatible TiO_2_/MoS_2_/PDA/RGD nanorod array on Ti implants, which could kill bacteria under dual light coirradiation and promoted the osteogenic capabilities of the implants. While the photothermal conversion ability of 660 nm is relatively weak, the yield of ROS under dual light irradiation is almost the sum of individual light irradiation, which enhanced the antibacterial ability of the implants. The physical puncture of the nanorods also contributed to the antibacterial properties of the implants. In addition, a novel photo-sonotherapy strategy has also been designed by fabricating an oxygen deficient S-doped TiO_2_ coating on Ti implants, which exhibited significant sonodynamic and photothermal abilities 15. This system achieved an extremely high antibacterial efficiency of 99.995% against *Staphylococcus aureus* under 15 min NIR light irradiation combined with ultrasound treatment. In addition, besides coating on the surface of bone implants, the NIR light-responsive nanomaterials could also be injected into the knee joint for the clinical treatment of periprosthetic joint infection. Liu *et al.* 93 has reported curcumin‑upconversion nanoparticles (curcumin-UCNPs) for the efficient eradication of drug-resistant bacteria in a deep joint infection by producing singlet oxygen under NIR light irradiation. Curcumin is an extract from the plant *Curcuma longa*, which has intrinsic antibacterial and anti-inflammatory properties. Compared with traditional therapeutic strategies, this research might offer a new alternative for periprosthetic joint infection.

In summary, the NIR light-assisted phototherapies have shown stable antibacterial photothermal and photodynamic therapeutic performances* in vitro* and* in vivo*, representing promising strategies to treat bone infections.

#### NIR light-assisted phototherapy for arthritis

Worldwide, the prevalence of arthritis is high, and includes over 100 types, of which the most common are rheumatoid arthritis, osteoarthritis, and inflammatory arthritis 94. NIR light has sufficient penetration depth to reach the deep inflamed joints, which guarantees the efficacy of phototherapies and desirable therapeutic outcomes. NIR light-responsive nanomaterials could exhibit outstanding photothermal and photodynamic therapeutic effects for the anti-inflammation therapy via intra-articular injection, which is similar to the mechanisms in anticancer and antibacterial therapies. These photothermal materials could convert NIR light into localized heat and suppress the inflammatory reaction. In parallel, the photosensitizers in PDT can also be excited into their singlet state under NIR light irradiation, producing ROS to treat inflamed cells and tissues 83, 95. These synergistic photothermal and photodynamic effects of NIR light-assisted phototherapies might decrease the synovium erosion, chronic inflammation activity, and bone and cartilage destruction, providing a promising approach for arthritis treatment 95.

Rheumatoid arthritis (RA) is a common chronic inflammatory disease that seriously affects the quality of life of the patients, leading to long standing synovitis, degradation of cartilage and bone tissue, and increased disability. Recently, NIR light‑assisted phototherapy including PTT and PDT, has received increased attention for noninvasive and spatiotemporal controlled RA treatment 96-98. Nanoparticles with good biocompatibility, excellent photoresponsive ability, and intrinsic osteogenic/chondrogenic capabilities have significant advantages for RA phototherapies. Lu *et al*. 95 prepared novel _L_-cysteine (Cys) assisted Cu_7.2_S_4_ nanoparticles to serve as photothermal agents for PTT and photosensitizers for PDT simultaneously. This kind of nanoparticle can be injected into the disease region and has full potential for RA treatment. Meanwhile, a novel therapeutic system that combined NIR light-responsive BP nanosheets with a platelet-rich plasma (PRP)‑modified chitosan thermoresponsive hydrogel has been prepared to treat RA 83. The BP nanosheets generate mild local hyperthermia upon NIR light irradiation and release ROS into the inflamed joints simultaneously to eliminate hyperplastic synovial tissue. In addition, the degradation products of BP enhance the osteogenic process and the thermoresponsive hydrogel can serve as an anti‑inflammation drug carrier, reducing the friction between the surrounding tissues at the same time. These injectable NIR light-responsive systems can be used as efficient alternatives to anti-inflammation therapies for RA. The above-mentioned NIR light‑responsive phototherapy systems for RA are summarized in **Table 5.**

Moreover, NIR light-triggered drug delivery systems have been developed for arthritis treatment. As NIR light-triggered drug delivery systems for bone cancer chemotherapies, the photothermal and photodynamic mechanisms of NIR light could also be used to achieve drug release in arthritis treatment. Zhao *et al*. 99 reported a highly efficient photothermal-triggered drug delivery platform based on the molybdenum disulfide (MoS_2_) nanosheets to control the release of dexamethasone for osteoarthritis therapy. This nanosystem could be used to treat osteoarthritis *in vivo* through intra-articular injection and the NIR light could control dexamethasone release remotely in the disease region, which could be modulated by adjusting the light‑radiation behavior. Similarly, cell membrane penetrating peptide‑modified mesoporous silica nanoparticles (MSNs) were reported to realize the efficient controlled release of triptolide, which is a traditional Chinese herbal medicine used to treat RA 100. The MSNs modified with indocyanine green, a kind of photothermal agent, could effectively regulate the release of triptolide under 808 nm laser irradiation, leading to the development of noninvasive therapeutic approaches for RA. These strategies could serve as references for the design of other intra-articular drug delivery nanosystems and have marked potential for the clinical treatment of arthritis.

### NIR light-assisted phototherapy for bone tissue regeneration

**Figure 6:**
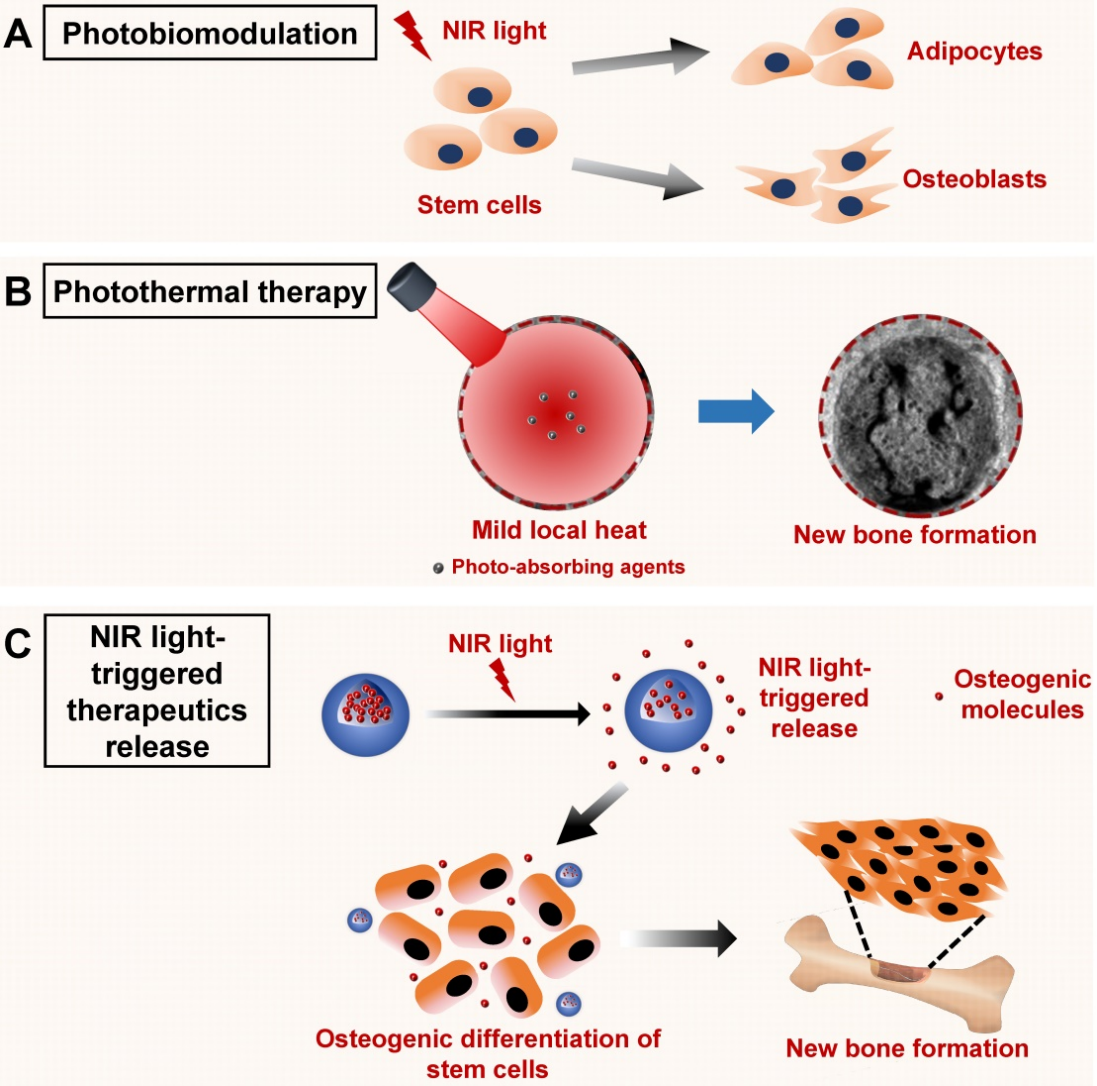
Schematic illustration of the NIR light-induced photobiomodulation, photothermal effect, and NIR light-triggered osteogenic molecule release systems to regulate new bone formation. **A.** Photobiomodulation therapy under NIR light irradiation can modulate the attachment, proliferation, and differentiation of stem cells. **B.** Phototherapy based on the NIR light-responsive nanomaterials can induce mild local heat under NIR light irradiation, which enhances new bone formation. **C.** NIR light‑responsive release systems can realize the controlled release of osteogenic molecules, such as ions, drugs, and cytokines, to accelerate new bone formation. NIR, near infrared.

Recently, several strategies to accelerate bone tissue regeneration via NIR light‑assisted phototherapies have been proposed. The mild localized heat or photoelectric microenvironment triggered by NIR light irradiation can achieve noninvasive, remote, and spatiotemporally controlled cell differentiation behaviors, providing a unique strategy for bone tissue regeneration 19, 20. In addition, considering that NIR light-responsive biosystems have been applied to realize the remote and controlled release of anticancer or antibacterial drugs, it also could be used to facilitate the controlled release of biological molecules (such as ions, drugs, or proteins) to promote bone tissue regeneration or promote tissue regeneration via a nonpharmacological and noninvasive strategy. At the same time, it is suggested that photobiomodulation therapy under NIR light irradiation could have a positive effect on bone metabolism, providing more inspirations for clinical applications in bone‑related disease treatment. At the cellular level, stem cells and progenitor cells appear to be particularly susceptible to photobiomodulation effects. NIR light-induced photon absorption mainly occurs in mitochondria. Numerous signaling pathways could be activated via the promoted oxidative metabolism within the mitochondria, leading to the activation of a series of transcription factors 101. These transcription factors can lead to increased expression of genes that modulate the attachment, proliferation, and osteogenic differentiation of cells, playing important roles in accelerating wound healing and bone tissue regeneration 102. Therefore, NIR light-assisted phototherapies have several advantages for bone tissue regeneration (**Figure 6**). These applications are summarized in the following sections.

#### Phototherapy based on NIR light-responsive nanomaterials for bone tissue regeneration

Controlling cell fate through noninvasive *in vitro* stimulation can achieve precise and orchestrated biological activities in tissue engineering. It represents an appealing strategy to use NIR light radiation as a kind of tissue penetrative stimuli to gain nonpharmacological and noninvasive control of cell differentiation behaviors. For example, Fu *et al*. 20 designed a bismuth sulfide/hydroxyapatite (BS/HAp) film to build a rapid and repeatable NIR light-sensitive photoelectric extracellular microenvironment around Ti implants. The photoelectrons created under 808 nm laser radiation could tune the behavior of MSCs and control cell fate by regulating downstream gene expression towards osteogenic differentiation. This BS/HAp modification on an implant is highly encouraging and could achieve more precise control of cell fates in biological therapies. Similarly, Tiwari *et al.* reported a kind of NIR light-absorbing carbon nitride (C_3_N_4_) sheet, which enhances cellular proliferation and differentiation through runt-related transcription factor 2 (RUNX2) activation. The photocurrent upon two photon excitation of this small sized C_3_N_4_ sheet would induce a charge transfer and increased cytosolic Ca^2+^ accumulation, resulting in enhanced osteogenic differentiation of stem cells and new bone formation. In addition, single‑walled graphene nanoribbons have been reported to generate photoacoustic signals under 905 nm NIR laser radiation, which could serve as an anabolic stimulus for noninvasive bone defect repair in a rodent femoral fracture site 103.

By contrast, mild local heat (40-43 °C) has been reported to facilitate healing of bone defects via inducing the earlier differentiation of human MSCs (hMSCs) and promoting the maturation of osteoblasts differentiated from MSCs 104. Several studies have reported the application of NIR light-triggered photothermal technology in bone tissue engineering. For example, classical NIR light responsive nanomaterials, such as metal nanoparticles, could serve as hyperthermal agents to promote bone tissue regeneration by producing mild localized heat. A kind of NIR light-responsive gold nanorod doped gelatin/HAp composite microsphere was successfully synthesized, which exhibited an efficient NIR light-triggered photothermal properties 105. Zhang *et al*. 19 reported a photothermal strategy for *in situ* bone regeneration using porous AuPd alloy nanoparticles (pAuPds) as photothermal agents. The mild localized heat (around 40-43 °C) could greatly enhance cell proliferation and bone tissue regeneration via upregulating the expression of a series of osteogenesis-related genes and proteins. RNA sequencing analysis demonstrated that the Wnt signaling pathway was involved in *in situ* bone regeneration. Moreover, a new class of 2D BP nanosheets have been employed as photothermal agents for the remote control of bone regeneration 106, 107. These BP nanosheets could upregulate the expression of heat shock proteins in human BMSCs and thus enhance bone tissue regeneration by remote control of NIR light irradiation. Moreover, BP can degrade into nontoxic phosphates and phosphonates in the biological environment, providing good nucleation sites for biomineralization 108, 109. The BP-combined osteoimplants have good photothermal properties, biodegradability, and osteogenic abilities, thus providing new insights for orthopedic applications.

#### NIR light-responsive release systems for bone tissue regeneration

Although the use of NIR light-responsive drug delivery systems combining chemotherapy and photothermal therapy has exhibited versatility in bone cancer therapies, NIR light‑responsive release systems for bone tissue regeneration have not been widely used. NIR light has good tissue penetration, as well as accurate and spatiotemporal control properties. Remote control of cell fate could provide a versatile method to regulate bone regeneration therapies. Combining the use of tissue penetrative NIR light stimuli with NIR light-responsive nanomaterials is a delicate and efficient strategy for the intracellular control of stem cells. Recently, several strategies have been proposed to regulate the differentiation of stem cells based on NIR light-responsive release nanosystems. Kang *et al*. 110 designed an upconversion nanotransducer (UCNT)‑based nanosystem to regulate intracellular calcium by encapsulating either a calcium chelator or a calcium supplier under NIR light stimuli. An increase in calcium can promote the differentiation of hMSCs into osteoblasts. Strontium (Sr) is another osteoinductive element that could enhance bone formation. An NIR light-triggered Sr^2+^ delivery system was developed by incorporating SrCl_2_ and BP nanosheets into poly(lactic-co-glycolic acid) (PLGA) microspheres for bone tissue regeneration 29. The spatiotemporal release of Sr^2+^ controlled by NIR light significantly improved bone tissue regeneration. These NIR light-triggered ion-release systems are suitable for bone regeneration therapies that require precise control at specific times.

Alternatively, the on-demand release of therapeutic proteins holds great promise for bone tissue regeneration. Tuncaboylu *et al*. 111 designed a shape-memory poly(ɛ-caprolactone) network to release several proteins, including stromal cell-derived factor 1α (SDF-1α), a chemotactic protein relevant to bone tissue regeneration. The diameter of these shape-memory tubes decreases under NIR light irradiation and expels the payload protein. Yin *et al*. 22 provided another strategy to fabricate a biomimetic anti‑inflammatory nanocapsule (BANC). The BANC is coated with lipopolysaccharide-treated macrophage cell membranes, loading resolvin D1 inside a gold nanocage as an “M2 polarization inducer”. Controlled release could be triggered under NIR light irradiation, promoting the healing process during bone tissue repair. Moreover, another heat-activated and dimerizer-dependent transgene expression system was designed based on an NIR light-responsive hydrogel to achieve spatiotemporal control of bone morphogenetic protein-2 (BMP-2) 21. The photothermal effect of hollow gold nanoparticles in hydrogels induced mild hyperthermia, which stimulated the cell constructs to express BMP-2 in the bone defect. These protein-release systems promote the formation of new mineralized bone tissue, providing promising strategies for bone tissue regeneration. These NIR light‑responsive drug release systems are summarized in **Table 6.**

#### Photobiomodulation therapy under NIR light irradiation for bone tissue regeneration

Photobiomodulation describes the photochemical reactions of cells and tissues achieved under red or NIR light irradiation with lower energy densities compared with other heat-mediated laser therapies, which induce tissue ablation or coagulation after irradiation 102, 112. This light-based technique involves irradiation of tissue using lasers or LEDs. Laser light can reach the target tissue regions via a continuous or pulsed mode. The transmitted energy will trigger photochemical reactions instead of inducing a thermal effect, which can modulate cellular metabolic processes, including cell viability, proteins synthesis, and DNA/RNA expression 113. Currently, photobiomodulation therapy under NIR light irradiation has been used clinically to reduce inflammation, edema, and pain, and to repair multiple damaged tissues, representing a promising alternative for bone disease treatment and bone tissue regeneration 112, 114. For photobiomodulation therapy, two critical challenges must be overcome: One is the uncertainty about the mechanism underlying its beneficial effects, and the other is the optimal irradiation parameters 12. In the following sections, the effects of photobiomodulation therapy on cellular behavior, the bone healing process, and dental treatments are discussed based on the published literature.

##### The effects of photobiomodulation therapy on cellular behavior

Stem cell therapy has attracted great interest in tissue regenerative medicine. Photobiomodulation therapy might increase the biological functions of MSCs. The primary action site of photobiomodulation under red or NIR light irradiation is the mitochondria, more specifically, cytochrome c oxidase. It is hypothesized that inhibitory nitric oxide dissociated from cytochrome c oxidase could restore electron transport and increase the mitochondrial membrane potential. By contrast, NIR light irradiation could trigger light or heat-gated ion channels and activate the change of transient receptor potential. These potential changes to mitochondria could increases the rate of respiration and ATP production, leading to a marked effect on stem cells via mitochondrial redox signaling 112. Our literature review showed that the effect of photobiomodulation therapy for bone tissue regeneration not only depends on the type of cells, but also on the category of lasers, wavelength, density of energy, and the duration of photobiomodulation therapy. There is a wide variation in the parameters between different studies, making it difficult to compare the efficacy of photobiomodulation. Different types of cells, such as adipose-derived stem cells (ASCs) and BMSCs, might have different responses to photobiomodulation therapy 112. It has been reported that photobiomodulation therapy with a combination of 630 and 810 nm lasers irradiation significantly enhanced cell viability and decreased the apoptosis of human ASCs and BMSCs *in vitro*, in which NIR light irradiation induced more *in vitro* cell viability of ASCs than BMSCs 115.

In addition, the effects of photobiomodulation therapy within different wavelengths are still being explored. Wavelengths from blue to NIR light have been demonstrated to enhance bone regeneration. Kunimatsu's group have reported that irradiation with a high frequency NIR diode laser is highly efficient to increase cell division and migration of osteoblasts via MAPK/ERK signaling and improved the bone tissue regeneration 116. Under 1064-nm irradiation, equine MSCs remained viable and expressed increased concentrations of interleukin (IL)-10 and vascular endothelial growth factor (VEGF), which enhanced the tissue healing process by decreasing the inflammatory reaction and promoting angiogenesis 117. The cellular responses to NIR light irradiation demonstrate the great potential for the photobiomodulation effect on MSCs to enhance their therapeutic properties. Meanwhile, it has been reported that low-power irradiation by 635 and 809 nm lasers might not have obvious effects on osteoblast viability and proliferation 114. Moreover, Wang's group have demonstrated that although red (660 nm) or NIR (810 nm) photobiomodulation therapies stimulated the proliferation of hASCs, the blue (415 nm), green (540 nm) wavelengths are more effective to stimulate the osteogenic differentiation of hASCs 118, 119. Similarly, Tani *et al*. 120 reported that photobiomodulation therapy with a 635 nm laser was a more effective option to enhance the viability, proliferation, adhesion, and osteogenic differentiation of human MSCs compared with those of 808 nm and 405 nm irradiation. Therefore, the most promising range of wavelengths to promote the proliferation of osteoblasts and tissue regeneration is still controversial 114. It is important to use appropriate irradiation parameters during laser treatment for bone tissue engineering.

Moreover, guiding the differentiation of MSCs by light is particularly attractive for bone tissue engineering and regenerative medicine. Yan's group constructed a controlled UCNPs-based substrate to guide multi-directional differentiation of MSCs by NIR light irradiation 121. Under NIR light irradiation of different power densities, MSCs tend to differentiate into adipocytes (low power, 0.5 W·cm^-2^) or osteoblasts (high power, 6 W·cm^-2^). The NIR light triggered anti-adhesive effect of a 4‑(hydroxymethyl)‑3-nitrobenzoic acid modified poly(ethylene glycol) coating on a substrate demonstrated these differentiation properties of MSCs, providing a new way to modulate bone regeneration in tissue engineering.

##### The photobiomodulation therapy under NIR light irradiation for bone healing

Several studies have demonstrated that NIR light irradiation therapy could be used to improve bone healing and the regeneration process. NIR light therapy at 790 nm was reported to guide bone tissue regeneration on rabbits' tibial fractures with wire osteosynthesis or internal rigid fixation 122-124. In addition, 850 nm NIR light phototherapy demonstrated the same ability to stimulate cell proliferation and guide bone tissue regeneration in a rodent model 125. Moreover, linear polarized NIR light irradiation (830 nm) could enhance the mechanical expansion of the rat sagittal suture by controlling the growth of the craniofacial skeleton 126. These NIR light irradiations showed deep penetration in the bone defect region and stimulated bone healing, resulting in an increased deposition of calcium hydroxyapatite in the radiation regions 122.

Furthermore, Gokmenoglu *et al*. 127 reported that photobiomodulation therapy under NIR light irradiation could change the biochemical markers in peri-implant cervical fluid and the biological activities in the wound healing process. In that study, an LED device at a wavelength in the NIR region was applied to the surgical region, leading to a positive effect on the osseointegration process and implant stability after surgery. Similarly, the effect of LED (945 nm) therapy on the bone healing and tissue regeneration processes has also been investigated 128. These studies demonstrated that low power LED therapy within the NIR region has a positive effect on tissue regeneration, especially in the early stage of repair. The results from those studies have shown that NIR light irradiation can accelerate the process of fracture healing and promote the formation of new bone tissue, providing great potential for the clinical treatment of bone fractures.

##### The photobiomodulation therapy under NIR light irradiation for dental treatment

Tooth movement during orthodontic therapy is usually associated with bone resorption on the pressure side and bone regeneration on the tension side 129. Several strategies have been proposed to decrease the treatment time in orthodontic therapy. A multicenter clinical trial demonstrated that photobiomodulation under 850 nm NIR light changed the rates of tooth movement and promoted orthodontic alignment significantly in the early phase of treatment 130. NIR light irradiation at a power density of 60 mW·cm^-2^ was performed on the surface of the patients' cheek for 20 or 30 min every day or 60 min every week. Another study supported this conclusion, providing evidence that low level laser therapy accelerates bone remodeling and inhibits root resorption during orthodontic tooth movement in a rodent model 131. The laser irradiation (810 nm, 5 W·cm^-2^) in that study was performed on the labial and palatal sides of the tooth for 15 s every other day. Moreover, Gunji *et al*. 129 examined the effect of high frequency NIR diode laser irradiation with a wavelength of 910 nm on tooth movement. They reported that 910 nm NIR laser irradiation on periodontal tissue resulted in metabolic activation, which ultimately enhanced tooth movement during orthodontic treatment. Recently, the combined effects of mechanical force loading and Nd:YAG laser irradiation on bone metabolism have been investigated 132. NIR laser irradiation at a wavelength of 1064 nm activated the expression of genes related to bone metabolism in osteoblast‑like cells, enhancing bone remodeling and tooth movement.

Besides the reported capability to enhance tooth movement, NIR light-assisted phototherapy has also been applied to regulate the inflammatory and repair processes of dental trauma. LED phototherapy (940 nm) of the periodontal ligament stimulated the proliferation of fibroblasts and increased the synthesis of the extracellular matrix, accelerating periodontal tissue repair 133. Irradiation could also decrease the migration of inflammatory cells into periodontal tissues, thus reducing root resorption. Another study demonstrated that 810 nm laser phototherapy on the root surface, in continuous or pulsed modes, could reduce root resorption during the treatment of tooth replantation 134. These studies suggested that NIR light‑assisted phototherapy might be efficient to decrease root resorption and enhance periodontal tissue healing after injury to periodontal tissue. However, Mitihiro *et al*. 135 reported that NIR LED (940 nm) phototherapy up to 48 h after tooth replantation did not improve the healing process, and even delayed periodontal ligament repair. Although phototherapy seems to show a promising effect in decreasing the inflammatory reaction, it may not be effective to avoid pulp necrosis, which damages the process of tissue repair after tooth replantation. The results of our literature search literature suggested that caution should be taken when using NIR light-assisted phototherapy in dental treatment. Determining the irradiation parameters, such as the wavelength, power density, mode of emission (discontinuous or continuous), duration of irradiation, and the irradiated region, is essential for successful dental treatment.

## Current challenges and perspectives

In summary, NIR light can serve as an effective *in vitro* stimulation for targeted therapy because of its good tissue penetration ability, minimal invasiveness and high selectivity 50. NIR light-assisted phototherapies have shown great potential to treat bone-related diseases and bone regeneration. Most of these phototherapies are based on a series of unique NIR light-responsive nanomaterials. These nanomaterials could exert their functions via multiple methods administration according to different therapeutic goals. They could be injected directly, or serve as a part of bioactive scaffolds and drug delivery systems, exhibiting efficient therapeutic performance in the pathological regions via generating local hyperthermia and increasing ROS concentrations under NIR light irradiation. The different applications of NIR light‑responsive nanomaterials to treat bone-related diseases and bone regeneration are summarized in **Table 7.** However, the photobiomodulation effect under the direct NIR light irradiation has also shown great potential to regulate cellular behaviors and the process of bone tissue regeneration. To date, a series of *in vitro* and *in vivo* studies have demonstrated the encouraging advantages of the NIR light-assisted phototherapies for bone-related disease and bone regeneration. However, it remains challenging to translate preclinical studies into clinical applications.

Firstly, the biosafety of NIR light-responsive nanomaterials should be considered. With the rapid development of nanotechnology, several kinds of nanomaterials, such as metal-based nanomaterials, carbon-based nanomaterials, and other related photosensitive nanostructures, have been involved in NIR light-assisted phototherapies to make them “smart”. Apart from their efficient photosensitive properties and attractive biocompatibility, there are still many challenges to overcome before clinical applications. Above all, the components of these nanomaterials should be nontoxic and biodegradable. The acute and long-term toxicity of these nanomaterials depend on their physicochemical properties, including their composition, size, shape, and surface charge. The acute and chronic pathological toxicity during the treatment period of these nanomaterials, as well as their renal or hepatobiliary elimination from the body, should be evaluated to guarantee their* in vivo* safety for further clinical translation 32. In current phototherapies, most applied NIR light-responsive nanomaterials might have long-term toxicity, especially the nonbiodegradable inorganic nanoparticles. The nanomaterials with excellent biocompatibility and biodegradability, such as black and red phosphorus, are the preferred selections for various phototherapies. In addition, the size, shape and surface charge of the nanomaterials should be modified to minimize their cellular interactions with normal tissues, thereby avoiding nonspecific damage to normal cells. For example, the emerging MXenes have exhibited satisfactory photosensitive abilities, hydrophilic nature, and unique physiochemical properties, allowing them to meet the strict requirements of biomedicine. The large surface area and facile surface modification of the MXenes also endow them with better *in vivo* performance because of their reduced toxicity, enhanced colloidal stability, and extended circulation within the body 53, 54. Providing nanomaterials with a biological mimic coating, such as liposomes and cell membranes, could also be a promising alternative for clinical translation 11. In conclusion, these NIR light‑responsive nanomaterials should not induce any adverse effects and must receive Food and Drug Administration (FDA) approval before human use. Currently, few FDA‑approved phototherapeutic agents, such as indocyanine green, have been applied in NIR light-assisted phototherapies 136, 137. Increased efforts should be made to synthesize and design more nanomaterials with both high phototherapeutic efficiency and outstanding biocompatibility.

Secondly, NIR light-induced hyperthermia should be well controlled according to different therapeutic needs. The main side effect of NIR light-induced phototherapies for bone-related disease treatment is that local hyperthermia followed by NIR light irradiation can not only eliminate the cancer cells, bacteria, and inflammatory cells, but also might damage the normal cells and tissues when the temperature is above the threshold. The precise regulation of photothermal effect during the NIR light irradiation treatment is essential to promote the efficacy of phototherapies and minimize the damage. Considering the different therapeutic goals in the treatment of bone-related diseases, the parameters of NIR light irradiation to generate targeted hyperthermia within a reasonable temperature range should be carefully selected 21. Generally speaking, local hyperthermia over 50 °C exerts highly effective phototherapeutic effects on pathological tissues, which is favorable for the treatment of bone cancers, infections, and inflammations. Under NIR light irradiation, the cancer tissues can be effectively ablated by photonic hyperthermia (over 50 °C) without recurrence 31, 44, 66. Similarly, hyperthermia at almost 55 °C achieved a high antibacterial efficiency (99.995%) in the treatment of bone infections 15. For bone regeneration therapies, the proposed mild local hyperthermia (40-42 °C), which is only about 3-5 °C higher than the normal human body temperature (37 °C), is recommended because of its abilities to accelerate bone healing and enhance the mineralization process in bone tissue regeneration 106. Meanwhile, the temporary photothermal therapy within a suitable irradiation time might have a minimal impact on the tissue regeneration of the defect areas. The local inflammatory reaction secondary to PTT is similar to the primary phase of bone healing, which could accelerate bone tissue regeneration by recruiting precursor cells. Considering the effects of photobiomodulation on the adhesion, proliferation, and differentiation of stem cells, suitable mild local hyperthermia might have positive therapeutic effects on bone regeneration 44. In future studies, more accurate and sensitive real-time monitoring techniques should be developed to adjust the irradiation parameters and avoid unnecessary damage following overheating 11.

Furthermore, the administration of NIR light could also be optimized. Although NIR light has deeper tissue penetration than the UV and Visual light, more effort should be made to overcome the laser penetration problem for the treatments of deep‑seated bone cancer, infection, or inflammation. The optimization of excitation conditions and the improvement of nano-bio interactions are also important. Local implantation and injection, rather than intravenous injection, could deliver effective amounts of photosensitive nanomaterials to targeted sites to obtain better therapeutic outcomes. Recently, phototherapy using an optical fiber NIR laser device was combined with surgery, in which the deep pathological lesions could be directly exposed to laser irradiation 138. These emerging techniques using light administration could promote the efficiency of NIR light phototherapy, and advance its further translation into clinical applications.

In conclusion, NIR light-assisted phototherapies have shown great potential to treat bone-related disease and bone tissue regeneration. This noninvasive, precisely targeted treatment strategy could be considered as an effective tool to promote therapeutic efficacy. With better understandings of this technology, NIR light-assisted phototherapy could serve as a promising alternative for clinical applications.

## Figures and Tables

**Table 1:** NIR light-responsive biomaterials for bone cancer phototherapy

Phototherapies	Photoresponsive agents	Wavelength (nm)	Power density	Radiation time (min)	*In vitro* phototherapeutic effects	*In vivo* phototherapeutic effects	References
PTT	Nanographene oxide sheets	808	1.5 W·cm^-2^	5	Effective decrease of osteosarcoma cell viability	-	Cicuéndez M. *et al*. 47
	Multi-walled carbon nanotubes (MWNTs)	808	1.25 W·cm^-2^	1	Effective decrease of breast cancer cell viability to 74.3%	Significantly suppressed growth of breast cancer bone metastasis	Lin Z. *et al*. 49
	Platinum nanoparticles	808	3.5 W·cm^-2^	7	Obvious G1 arrest in cancer cells	Efficient bone-targeted anticancer activity	Zhou Z. *et al*. 48
			2.5 W·cm^-2^	10	Effective decrease in tumor cell viability	Effective inhibition of tumor growth in a bone metastasis model	Wang C. *et al*. 50
	Na_x_WO_3_ nanoparticles	980	1.5 W·cm^-2^	5	Remarkable decrease in breast cancer cell viability	Significant decrease in tumor volume	Jie S. *et al*. 51
	2D Nb_2_C nanosheets	808	1.0 W·cm^-2^	5	Significant photothermal tumor cell ablation and endocytosis	Highly efficient photothermal bone tumor eradication	Lin H. *et al*. 32
		1064					
	Carbon dot/WS_2_ nanosheets	1064	0.6 W·cm^-2^	5	Effective decrease of human osteosarcoma cell viability	Complete eradication of osteosarcoma without reoccurrence	Geng B. *et al*. 42
PDT	Pd-bacteriopheophorbide	650-808	360 J/cm	30	-	Complete tumor elimination in 50%	Koudinova N. V.* et al*. 41
			54 J/0.6 cm				
	Black phosphorus	650-808	-	15	Inhibited metabolic activity of osteosarcoma cells	-	Raucci M.G. *et al*. 58

PTT: Photothermal therapy; PDT: Photodynamic therapy.

**Table 2:** NIR light-responsive scaffolds for bone cancer phototherapy

Photoresponsive agents	References	Scaffolds	Irradiation condition			Step 1 mechanism:	Step 2 mechanism:	*In vivo* phototherapeutic effects: Bone tumor bearing model of nude mice	*In vivo* bone tissue regeneration	
			Wavelength (nm)	Power density (W·cm^-2^) and Radiation time	Highest tissue temp. (°C)					
						Anti-cancer	Bone tissue regeneration	(cell type, observation time)	(animal models, observation time)	
Bismuth (Bi)	Wang L. *et al*. 60	Bi-doped BG scaffolds	808	1.5 W·cm^-2^, 10 min	55	Photothermal effect of Bi	Osteoconductivity and osteoinductivity of BG	Complete elimination at day 3 (rat osteosarcoma cells, 15 days)	NM	
Hydrogenated black TiO_2_ (H-TiO_2_)	Zhang W. *et al*. 61	Hydrogenated black TiO_2_ coating Ti6Al4V implant	808	0.4 W·cm^-2^, 10 min, 2 days after implantation	52	Photothermal effect of H-TiO_2_	Osteogenesis abilities of H-TiO_2_ coating with hierarchical micro/nano-topographies	86.77% tumor cell necrosis rate (Saos-2 cells, 14 days)	Enhanced cellular adhesion, spread, proliferation, and osteogenic differentiation of rBMSCs (*in vitro* studies)	
Fe_3_O_4_	Zhao P. *et al*. 62	GdPO_4_/Chitosan/Fe_3_O_4_ scaffolds	808	4.6 W·cm^-2^, 3 min for 14 days	45.4	Photothermal effect of Fe_3_O_4_ nanoparticles	Osteogenesis abilities of as-released Gd^3+^ ions	Significantly reduced tumor growth (human breast cancer bone metastasis tumor cells, 14 days)	BV/TV: 61.23 ± 2.12% (calvarial-defect model of SD rats, 12 weeks)	
SrFe_12_O_19_	Yang F. *et al*. 63	Multifunctional magnetic mesoporous calcium silicate/chitosan porous scaffolds	808	0.3 W·cm^-2^, 6 min post-implantation	44	(a) photothermal effect of SrFe_12_O_19_, (b) NIR triggered DOX release	Osteogenesis abilities of magnetic nanoparticles and calcium silicate	Significantly decreased tumor growth (osteosarcoma MNNG cells, 12 days)	More newborn bone formation, BV/TV: 57.32 ± 3.53% (calvarial-defect model of SD rats, 12 weeks)	
	Lu J. *et al*. 64	Magnetic SrFe_12_O_19_ nanoparticles modified-mesoporous bioglass /chitosan porous scaffold	808	0.3 W·cm^-2^ , 6 min	43	Photothermal effect of SrFe_12_O_19_ nanoparticles	Osteoconductivity of bioglass and SrFe_12_O_19_ nanoparticles	Tumor cell necrosis rate: 84.6% (osteosarcoma MNNG cells, 12 days)	More newborn bone formation, BV/TV: 63±4% (calvarial-defect model of SD rats, 24 weeks)	
Cu-TCPP	Dang W. *et al.* 65	Cu-TCPP-TCP composite scaffolds	808	0.9 W·cm^-2^, 10 min every day in the first week, once every two days in the second week	55	Photothermal effect of Cu-TCPP nanosheets	Osteogenesis abilities of constituent elements(Cu, Ca and P)	Effective ablation and restrained growth of tumor (Saos-2 cells, 18 days)	Excellent bone-forming bioactivity (femoral defect model of New Zealand white rabbits, 8 weeks)	
CuFeSe_2_	Dang W. *et al*. 43	CuFeSe_2_ nanocrystals integrated 3D printed BG scaffolds	808	0.55 W·cm^-2^, 10 min every day in the first week, every 2 days in the second week	52	Photothermal effect of CuFeSe_2_ nanocrystals	Osteogenesis abilities of constituent elements (Cu, Fe, Se, Ca, Si, P)	Tumor cell necrosis rate: 96% (Saos-2 cells, 14 days)	Good bone-forming capacity (femoral defect model of New Zealand white rabbits, 8 weeks)	
MoS_2_	Wang H. *et al*. 66	MoS_2_/PLGA coating BG scaffolds	808	2 W·cm^-2^, 10 min at day 1, 2, 4 and 8	NM	Photothermal effect of MoS_2_ nanosheets	Osteogenesis abilities of constituent elements (Mo, Si, Ca, P)	Decreased tumor size to 15% (human osteosarcoma cells, 14 days)	Excellent osteogenic ability (calvarial-defect model of SD rats, 3 months)	
DTC cocrystal	Xiang H. *et al*. 45	DTC@3D printed BG scaffolds	808	1.5 W cm^-2^, 10 min	55	Photothermal effect of DTC	Osteoconductivity and osteoinductivity of BG	Promoted substantial tumor suppression (Saos-2 cells, 2 weeks)	Better newborn bone formation BV/TV: 43.5 ± 2.7% (calvarial-defect model of SD rats, 8 weeks)	
Graphene oxide (GO)	Xu C. *et al.* 71	Tricalcium silicate/GO bone cement	808	0.66 W·cm^-2^,10 minutes every two days	55	Photothermal effect of GO	Osteogenesis abilities of constituent elements (Ca, Si)	Decreased tumor volume, progressive apoptosis, and necrosis (Saos-2 cells, 14 days)	Enhanced cellular adhesion, spread, proliferation, and osteogenic differentiation of MC3T3-E1 cells (*In vitro* studies)	
	Ma H. *et al*. 59	GO/TCP 3D printed scaffold	808	0.42 W·cm^-2^ , 10 min	50		Osteoconductivity and osteoinductivity of TCP	Tumor cell necrosis rate: 83.28% (osteosarcoma MG-63 cell, 15 days)	Promoted bone-forming bioactivity (calvarial-defect model of New Zealand white rabbits, 8 weeks)	
Carbon dots (CD)	Lu Y. *et al*. 68	Zero-dimensional CD doped chitosan/ nanohydroxyapatite scaffold	808	1.0 W·cm^-2^, 10 min	51.4	Photothermal effect of CD	Osteoconductivity and osteoinductivity of nanohydroxyapatite	Effective suppression of tumor growth (UMR-106 osteosarcoma cells, 14 days)	Enhanced osteoinductivity (gluteus maximus muscle pouch of SD rats, 4 weeks)	
MXene	Yang Q. *et al.* 44	2D mesoporous silica@Nb_2_C MXene-integrated 3D printed BG scaffolds	1064	1.0 W·cm^-2^, 10 min, 1 day after implanting	50	(a) Photothermal effect of Nb_2_C MXene,(b) NIR triggered NO release	Phosphorus and calcium components degraded from BG	Complete elimination without recurrence (Saos-2 cells, 2 weeks)	Excellent osteogenic performance (calvarial-defect model of SD rats, 24 weeks)	
	Pan S. *et al.* 31	2D Ti_3_C_2_ MXene integrated 3D printed BG scaffolds	808	1.0 W·cm^-2^, 10 min, 1 day after implanting	63	Photothermal effect of Ti_3_C_2_ MXene	Osteoconductivity and osteoinductivity of BG	Complete elimination without recurrence (Saos-2 cells, 2 weeks)	Better newborn bone formation (calvarial-defect model of SD rats, 24 weeks)	
LaB_6_	Dang W. *et al*. 74	LaB_6_ micro-nanoparticles/PDLLA-modified β-TCP scaffolds	808	0.7 W·cm^-2^, 10 min every day for a week	53	Photothermal effect of LaB_6_ micro-nanoparticles	Osteoconductivity and osteoinductivity of B element and β-TCP	The inhibition of tumor growth (Saos-2 cells, 18 days)	More newborn bone formation (femoral defect model of New Zealand white rabbits, 8 weeks)	

BG: Bioactive glass, NM: Not mentioned, rBMSCs: Rat bone mesenchymal stem cells, BV/TV: New-bone volume/tissue volume, SD rats: Sprague Dawley rats, DOX: Doxorubicin, Cu-TCPP: Copper coordinated tetrakis (4-carboxyphenyl) porphyrin, TCP: Tricalcium phosphate, PLGA: Poly(lactic-co-glycolic acid), DTC: Dibenzotetrathiafulvalene tetracyanobenzene cocrystal, NO: Nitric oxide, PDLLA: poly(_D,L_-lactide).

**Table 3:** NIR light-triggered drug delivery systems for bone cancer photo-chemotherapy

Nanocarriers structure	Photoresponsive agents	Radiation conditions			The mechanisms of phototherapeutic effects	Anticancer drugs	Encapsulation efficiency	Release efficiency	Application	References
		Wavelength (nm)	Power density (W·cm^-2^)	Radiation time (min)						
Bismuth sulfide (Bi_2_S_3_)@mesoporous silica nanoparticles (MSNs)	Bi_2_S_3_	808	1	10	The outstanding photothermal conversion efficiency of Bi_2_S_3_ nanoparticles	DOX	99.85%	Almost 30%	Osteosarcoma	Lu Y. *et al*. 46
PDA coated bioactive glass nanoparticle (BGN)	PDA	808	1.4	10	The stable NIR light-excited photothermal effects of PDA coating	DOX	59%	Almost 10% (50^th^ day, pH = 5.5)	Bone cancer therapy and bone tissue regeneration	Xue Y. *et al*. 76
Gold nanorods enclosed inside mesoporous silica nanoparticles	Gold nanorods	808	1.2	10	The outstanding photothermal conversion efficiency of gold nanorods	ZOL	35.4%.	Almost 90%	Breast cancer bone metastasis	Sun W. *et al*. 77
PDA-alendronate /SN38 nanoparticles	PDA	808	3.6	30	Highly efficient photothermal effects of PDA nanoparticles	SN38	-	Over 50%	Malignant bone tumors and osteolysis	Wang Y. *et al*. 80
PDA-decorated nano-hydroxyapatite chitosan hydrogel	PDA	808	2	2	The excellent photothermal effects of PDA coating	Cisplatin	91.49%	Sustained release: 71% (9^th^ day)	Breast cancer bone metastasis and bone tissue regeneration	Luo S. *et al*. 81
Bovine serum albumin (BSA)-iridium oxide (IrO_2_) nanoparticles	IrO_2_	808	1	5	The superior photothermal conversion efficiency of IrO2 nanoparticles	DOX	27.4 wt%	46% (pH = 7.4), 68% (pH = 5.0)	Osteosarcoma	Gu W. *et al*. 82

DOX: Doxorubicin, PDA: Polydopamine, ZOL: Zoledronic acid, SN38: 7-ethyl-10-hydroxycamptothecin.

**Table 4:** Phototherapy based on NIR light-responsive biomaterials for implant-related infections

Reference	Surface modification	Photoresponsive agents	Antibacterial mechanism	Wavelength (nm) and, power density (W·cm^-2^)	*In vitro* studies		*In vivo* studies	
					Highest temperature (radiation time)	Antibacterial rates (*bacteria*, radiation time)	Highest temperature (position, radiation time)	Antibacterial rates (position, *bacteria*, radiation time)
Yuan Z. *et al.* 16	Mesoporous PDA nanoparticles (MPDA) + ICG + RGD coating	PDA	(a) accelerated bacterial death due to hyperthermia produced by photothermal conversion of MPDA, (b) ROS produced by released ICG with the assistance of heat	808 nm, 0.75 W·cm^-2^ *in vitro*, 2.0 W·cm^-2^ *in vivo*	51.2 °C (5 min),	99.7% (*S. aureus,* 5 min)*,*	51.3 °C (the femur of Sprague Dawley rats, 10 min),	95.4% (*S. aureus,* 10 min)
Yuan Z,* et al.* 139	MoS_2_/PDA-RGD coating	MoS_2_	(a) hyperthermia produced by photothermal effect, (b) accelerating GSH oxidation induced by NIR, (c) intrinsic ROS-independent oxidative stress of MoS_2_ nanosheets	808 nm, 0.5 W·cm^-2^ *in vitro*, 1 W·cm^-2^ *in vivo*	56.4 °C (8 min)	92.7 % (*S. aureus,* 8 min), 95.1 % (*E. coli,* 8 min)	51.5 °C (rabbits' tibia near keen joint, 10 min)	94.6% (*S. aureus*, 10 min)
Hong L. *et al*. 90	Bismuth sulfide (Bi_2_S_3_) @ trisilver phosphate (Ag_3_PO_4_) coating	Bi_2_S_3_	(a) hyperthermia produced by photothermal conversion of Bi_2_S_3_, (b) ROS produced by Bi_2_S_3_, (c) bacteriostatic properties of Ag_3_PO_4_	808 nm	52.4 °C within 3 min	0.5 W·cm^-2^ 3 min + 0.25 W·cm^-2^ 12 min: 99.45 % (*S. aureus*), 99.74 % (*E. coli*)	1.5 W·cm^-2^ + 1 W·cm^-2^ to maintain 50 °C (Sprague Dawley rats' tibia near the knee joint)	94.54% (*S. aureus*, 15 min)
Huang B. *et al.* 92	Red phosphorus/IR780/RGDC coating	Red phosphorus	(a) hyperthermia produced by photothermal conversion of red phosphorus, (b) ROS produced by IR780 under NIR light	808 nm, 0.5 W·cm^-2^	-	-	50 °C within 10 min (rats' tibia near keen joint)	Only a few bacterial colonies (*S. aureus*)
Tang L. *et al*. 28				808 nm, 0.5 W·cm^-2^ *in vitro*, 2.0 W·cm^-2^ *in vivo*	53.2 °C (250 s)	89.3 % (*S. aureus,* 10 min)	50 °C after 2 min of irradiation (rats' tibia near keen joint)	96.2% (*S. aureus*, 10 min)
Zhang G. *et al*. 27	TiO_2_/MoS_2_/PDA/RGD nanorod arrays coating	TiO_2_/MoS_2_	(a) ROS produced by 660 nm VL and 808 nm NIR light, (b) hyperthermia produced by photothermal conversion of MoS_2_ doped TiO_2_ NAs, and (c) physical puncture of the nanorods.	Dual light sources (808 nm, 0.5 W cm^-2^; 660 nm, 0.5 W·cm^-2^)	55 °C (10 min)	97.8% (*S. aureus,* 10 min)	50.2 °C (the back of Kunming mice, 10 min)	-
Su K. *et al.* 15	Oxygen-deficient S-doped TiO_2_ coating	Ti-S-TiO_2-x_	(a) ROS produced by 808 nm NIR light and ultrasound, (b) hyperthermia produced by photothermal conversion of Ti-S-TiO_2-x_	808 nm, 0.35 W·cm^-2^	54.9 °C (5 min)	99.995% (*S. aureus,* 5 min),	-	99.43% (the bilateral tibial plateaus of Wistar rats, *S. aureus,* 15 min)

PDA: Polydopamine, ICG: Indocyanine Green, RGD: Arginine-glycine-aspartic acid, ROS: Reactive oxygen species, GSH: Glutathione, RGDC: Arginine-glycine-aspartic acid-cysteine.

**Table 5:** NIR light-responsive phototherapy systems for rheumatoid arthritis

References	Phototherapy systems	Step 1: Phototherapy				Step 2: Osteogenesis	*In vivo* studies	
		Anti-inflammatory mechanisms	Wavelength, power density and radiation time	Photothermal effect	Photodynamic effect		Animal models	Phototherapy
Pan W.* et al*. 83	BPNs/Chitosan/PRP thermos-responsive hydrogel	Photothermal and photodynamic properties of BPNs	808 nm, 1.0 W·cm^-2^, 8 min	43.19% photothermal conversion efficiency	Distinct ROS generation	Biotherapy of PRP and phosphorus-driven, calcium-extracted biomineralization	DBA1/J mouse rheumatoid arthritis model	1.0 W·cm^-2^ 808 nm NIR light for 8 min
Lu Y. *et al*. 18	Cu_7.2_S_4_ nanoparticles	Photothermal and photodynamic properties of Cu_7.2_S_4_ NPs	808 nm, 1.0 W·cm^-2^, 10 min	55 °C in 500 µg·mL^-1^ Cu_7.2_S_4_ NPs solution	Singlet oxygen production and intracellular ROS generation	Osteogenic ability of Cu	CIA models (SD rats)	1.0 W·cm^-2^, 808 nm NIR light for 10 min

BPN: Black phosphorus nanosheets, PRP: Platelet-rich plasma, CIA models: Type II collagen induced rheumatoid arthritis models, UCNP: Upconversion nanoparticle, PJI models: Periprosthetic joint infection models.

**Table 6:** NIR light-responsive release systems for bone tissue engineering

Release systems	Photoresponsive agents	Wavelength (nm)	Power density	Osteogenic agents	Mechanisms	References
Photocaged UCNTs@mesoporous silica nanoparticles	Photocaged UCNTs	980	1 W·cm^-2^	Ca^2+^	Photocaged UCNTs regulate the intracellular calcium and stem cells differentiation by NIR light	Kang H. *et al*. 110
BP-SrCl_2_/PLGA microspheres	BP nanosheets	808	1 W·cm^-2^	Sr^2+^	NIR light triggers a local temperature rise and destroys the PLGA shells to release Sr^2+^	Wang X. e*t al*. 29
Poly(ε-caprolactone) (PCL) networks	IR-26 dye	1064	50 mJ	SDF-1α	NIR light triggers the shape switch of PCL networks to release SDF-1α	Tuncaboylu D. C. *et al*. 111
Biomimetic anti-inflammatory nanocapsule (BANC)	Gold nanocage	808	3 W·cm^-2^	RvD1	NIR triggers the release of RvD1 to induce the M2 polarization of macrophage cell	Yin C. *et al*. 22
Heat-activated and dimerizer-dependent transgene expression system	Hollow gold nanoparticles	808	11-17 mW·mm^-2^	BMP-2	Hyperthermia triggered by NIR laser activates the BMP-2 expression of cell constructs	Sanchez-Casanova S. *et al.* 21

UCNTs: Upconversion nanotransducer, BP: Black phosphorus, PLGA: Poly(lactic-co-glycolic acid), SDF: Stromal cell-derived factor, RvD1: Resolvin D1, BMP: bone morphogenetic proteins.

**Table 7:** Current applications of NIR light-responsive nanomaterials in bone-related diseases phototherapies

Applications	Administration of nanomaterials	Therapeutic mechanisms	Advantages	Drawbacks
Anticancer therapy	Direct intravenous injection	Photothermal and photodynamic properties of nanomaterials	Targeted accumulation in tumor sites, easy controllability	*In vivo* toxicity of non-degradable nanomaterials, unsuitable hyperthermia for normal cells as well as relatively complex design
	Implanted bone scaffolds	(a) photothermal and photodynamic properties of nanomaterials; (b) osteogenic capability of bioactive scaffolds	Efficient anticancer properties as well as osteogenic capability	
	Drug delivery systems (intravenous injection)	(a) photothermal and photodynamic properties of nanomaterials; (b) combined chemotherapeutic of anticancer drugs	Combined photo-chemotherapeutic effects	
Antibacterial therapy	Coating on the surface of bone implants	(a) photothermal and photodynamic properties of nanomaterials, (b) osteogenic capability of implants	Efficient noninvasive treatment of infection in deep tissue as well as simultaneous osteogenic activity	
Anti-inflammation therapy	Intra-articular injection	(a) photothermal and photodynamic properties of nanomaterials; (b) combined chemotherapeutic of anti-inflammation drugs, (c) osteogenic capability of co-delivery systems	Efficient NIR light induced anti-inflammation capability, controlled release of anti-inflammation drugs as well as simultaneous osteogenic activity	
Bone regeneration	Implantation	Mild local heat to promote bone regeneration	Controllable bone regeneration for precise medicine	Difficulty in controlling a suitable temperature
	Drug delivery systems (local injection)	Controlled release of osteogenic ions, drugs and proteins		

## Abbreviations

NIR: near infrared
PTT: photothermal therapy
PDT: photodynamic therapy
ROS: reactive oxygen species
NdYAG: neodymium-doped yttrium aluminum garnet
LED: light-emitting diodes
GO: graphene oxide
MWNTs: multi-walled carbon nanotubes
CDs: carbon dots
BP: black phosphorous
BMSCs: bone mesenchymal stem cells
DNA: deoxyribonucleic acid
BG: bioactive glass
Cu-TCPP: copper coordinated tetrakis (4-carboxyphenyl) porphyrin
TCP: tricalcium phosphate
2D: two dimensional
^1^O_2_: singlet oxygen
DOX: doxorubicin
PDA: polydopamine
BGN: bioactive glass nanoparticle
ZOL: zoledronic acid
ALN: alendronate
HAp: hydroxyapatite
*S. aureus*: *Staphylococcus aureus*
UCNPs: upconversion nanoparticles
RGD: arginine-glycine-aspartic acid
RGDC: arginine-glycine-aspartic-acid-cysteine
RA: rheumatoid arthritis
PRP: platelet-rich plasma
PA: photoacoustic
BS: bismuth sulfide
MSCs: mesenchymal stem cells
RUNX2: runt-related transcription factor 2
Sr: Strontium
PLGA: poly(lactic-co-glycolic acid)
SDF-1α: stromal cell-derived factor 1α
BANC: biomimetic anti-inflammatory nanocapsule
BMP-2: bone morphogenetic protein-2
ASCs: adipose-derived stem cells
hMSCs: human mesenchymal stem cells
CCO: cytochrome c oxidase
IL: interleukin
VEGF: vascular endothelial growth factor
